# Defect Passivation of 2D Semiconductors by Fixating Chemisorbed Oxygen Molecules via *h*‐BN Encapsulations

**DOI:** 10.1002/advs.202310197

**Published:** 2024-03-17

**Authors:** Jin‐Woo Jung, Hyeon‐Seo Choi, Young‐Jun Lee, Youngjae Kim, Takashi Taniguchi, Kenji Watanabe, Min‐Yeong Choi, Jae Hyuck Jang, Hee‐Suk Chung, Dohun Kim, Youngwook Kim, Chang‐Hee Cho

**Affiliations:** ^1^ Department of Physics and Chemistry Daegu Gyeongbuk Institute of Science and Technology (DGIST) Daegu 42988 South Korea; ^2^ School of Physics Korea Institute for Advanced Study (KIAS) Seoul 02455 South Korea; ^3^ International Center for Materials Nanoarchitectonics National Institute for Materials Science Tsukuba 305‐0044 Japan; ^4^ Research Center for Functional Materials National Institute for Materials Science Tsukuba 305‐0044 Japan; ^5^ Electron Microscopy and Spectroscopy Team Korea Basic Science Institute Daejeon 34133 South Korea; ^6^ Graduate School of Analytic Science and Technology Chungnam National University Daejeon 34134 South Korea

**Keywords:** chemisorption, defect passivation, hexagonal boron nitride, oxygen molecule, transition metal dichalcogenide

## Abstract

Hexagonal boron nitride (*h*‐BN) is a key ingredient for various 2D van der Waals heterostructure devices, but the exact role of *h*‐BN encapsulation in relation to the internal defects of 2D semiconductors remains unclear. Here, it is reported that *h*‐BN encapsulation greatly removes the defect‐related gap states by stabilizing the chemisorbed oxygen molecules onto the defects of monolayer WS_2_ crystals. Electron energy loss spectroscopy (EELS) combined with theoretical analysis clearly confirms that the oxygen molecules are chemisorbed onto the defects of WS_2_ crystals and are fixated by *h*‐BN encapsulation, with excluding a possibility of oxygen molecules trapped in bubbles or wrinkles formed at the interface between WS_2_ and *h*‐BN. Optical spectroscopic studies show that *h*‐BN encapsulation prevents the desorption of oxygen molecules over various excitation and ambient conditions, resulting in a greatly lowered and stabilized free electron density in monolayer WS_2_ crystals. This suppresses the exciton annihilation processes by two orders of magnitude compared to that of bare WS_2_. Furthermore, the valley polarization becomes robust against the various excitation and ambient conditions in the *h*‐BN encapsulated WS_2_ crystals.

## Graphical abstract

It is showed that *h*‐BN encapsulation greatly removes the defect‐related gap states by stabilizing the chemisorbed oxygen molecules onto the defects of monolayer transition metal dichalcogenide (TMD) crystals. By using electron energy loss spectroscopy (EELS) and optical spectroscopic measurements, this work unprecedentedly reveals the exact role of *h*‐BN encapsulation and the related excitonic properties in monolayer TMDs.

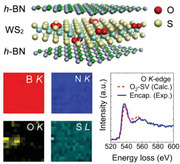

## 1 Introduction

Monolayer transition metal dichalcogenides (TMDs) have emerged as a platform to examine various exciton species such as trions, biexcitons, interlayer excitons, and moiré excitons due to the strong inter‐particle interactions.^[^ ^1^ ^−^ ^4^ ^]^ Furthermore, the valley‐dependent optical selection rules given by the broken inversion symmetry enable the selective generation of excitons in the particular valley (+K or −K) using circularly polarized light,^[^ ^5^ ^]^ providing the opportunity for applications toward valleytronic devices. However, the external disorders in close proximity of monolayer TMDs such as substrate‐induced surface roughness and absorbates can significantly alter the excitonic properties of two‐dimensional (2D) TMD materials, hindering the observation of the unique properties in monolayer TMDs.^[^ ^6^, ^7^ ^]^

In an attempt to reduce the disorder from substrates, it has been proposed to encapsulate TMD materials using hexagonal boron nitride (*h*‐BN) layers.^[^ ^8^, ^9^ ^]^ Recently, it has been shown that *h*‐BN encapsulation enables to observe the intrinsic optical properties of monolayer TMDs, including the excitonic linewidth with homogeneous broadening limit^[^ ^9^ ^]^ and the suppression of exciton annihilation processes.^[^ ^10^ ^]^ As the origin, the reduced substrate disorders have often been suggested in the previous works,^[^ ^9^, ^10^ ^]^ but the excitonic properties of TMDs on *h*‐BN substrates show large discrepancies from those of TMDs encapsulated by *h*‐BN.^[^ ^11^, ^12^ ^]^ On the other hand, previous investigations have shown that the oxygen molecules on the surface of TMDs also alter the electronic and optical properties of 2D TMD materials.^[^ ^13^ ^−^ ^18^ ^]^ The adsorption of the oxygen molecules occurs on the defects with relatively lower kinetic barrier rather than the perfect sites of TMD materials,^[^ ^18^ ^]^ and the oxygen molecules unlike other molecules can only be chemisorbed at chalcogen vacancies due to isovalent valence electrons (two unpaired electrons) with the chalcogen atom.^[^ ^14^, ^15^ ^]^ This chemisorption of oxygen molecules at the defect sites removes the defect‐related gap states without significantly altering the electronic band structures of TMD materials.^[^ ^13^, ^14^ ^]^ Thus, the oxygen molecules, that are supplied during the exposure of TMDs into the atmosphere, can significantly change the properties of defect‐related states through the chemical adsorption process. In this regard, *h*‐BN encapsulation can play a crucial role in the defect states of TMDs, in which the *h*‐BN layers fixate the adsorbed oxygen molecules on the TMD defects and facilitate the interaction between the oxygen molecules and the defect states. However, the role of *h*‐BN encapsulation in relation to the defects of TMDs remains unexplored.

In this work, we found that *h*‐BN encapsulation stabilizes the chemisorbed oxygen molecules on the defect sites of monolayer WS_2_ crystals, which greatly passivates the defect‐related gap states along with the decrease in the free electron density. Electron energy loss spectroscopy (EELS) combined with theoretical analysis clearly reveals that the oxygen molecules are chemisorbed onto the defects of WS_2_ crystals and are fixated by *h*‐BN encapsulation, that excludes a possibility of oxygen molecules trapped in bubbles or wrinkles formed at the interface between WS_2_ and *h*‐BN. Optical spectroscopic studies show that *h*‐BN encapsulation prevents the desorption of oxygen molecules over various excitation and ambient conditions, resulting in a greatly lowered and stabilized free electron density in monolayer WS_2_ crystals. This suppresses the exciton annihilation processes by two orders of magnitude compared to that of bare WS_2_. Furthermore, due to the stabilized free electron density in the *h*‐BN encapsulated WS_2_ crystals, the valley polarization becomes robust against the elevated excitation condition.

## 2 Results and Discussion

**Figure 1:**
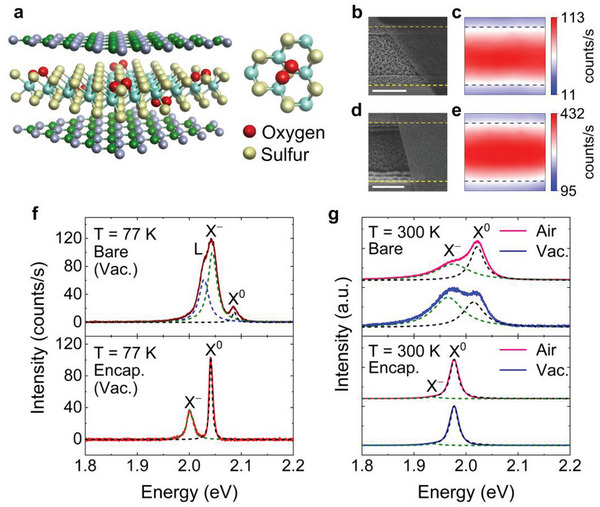
a) Schematic illustration showing the chemisorbed oxygen molecules anchored by the *h*‐BN encapsulation. Right inset image represents the detailed atomic configuration of the chemisorbed oxygen molecule at the sulfur vacancy. b) Scanning electron microscope image of the bare WS_2_ crystals on the line trenches. c) Spatial photoluminescence profile measured from the bare WS_2_ on the line trench. d) Scanning electron microscope image of the *h*‐BN encapsulated WS_2_ crystals on the line trench. e) Spatial photoluminescence profile measured from the *h*‐BN encapsulated WS_2_ on the line trenches. The yellow and black dashed lines marked in (b,d) and (c,e) indicate the boundary of the suspended and supported regions. The scale bars of (b,d) are 1 µm. f) Photoluminescence spectra for the bare (top panel) and *h*‐BN encapsulated (bottom panel) WS_2_ measured at a cryogenic temperature of 77 K under a vacuum level of ≈1 × 10^−5^ Torr. g) Photoluminescence spectra for the bare (top panel) and *h*‐BN encapsulated (bottom panel) WS_2_ measured under different ambient conditions of air and vacuum (T = 300 K). Each photoluminescence spectrum was fitted by Lorentzian functions. The black, olive, and blue dashed lines represent the neutral exciton (X^0^), trion (X^−^), and defect‐related trapped exciton (L) states, respectively.

**Figure** **1**a shows a schematic illustration showing that the fixated oxygen molecules by the *h*‐BN layers effectively passivate defects of the WS_2_. We considered the chemisorption type, where the oxygen molecule chemically bonds to three surrounding tungsten atoms, which is the most common configuration for the oxygen chemisorption (inset image of Figure 1a) in the S‐based TMDs.^[^ ^14^ ^−^ ^17^ ^]^ The chemisorbed oxygen molecules at the chalcogen vacancies can also be dissociated into two oxygen atoms, leading to a dissociative chemisorption, which occupies the sulfur vacancies with the dissociated oxygen atoms.^[^ ^13^ ^−^ ^15^ ^]^ However, in the case of the WS_2_ used in our study, the kinetic barrier for the O_2_‐chemisorption (0.56 eV) is lower than that for the O_2_‐dissociative chemisorption process (0.76 eV). It is estimated that the probability of the O_2_‐chemisorption is 1000 times higher than that of the dissociative chemisorpiton (see Figure S1, Supporting Information). Thus, the oxygen chemisorption on the monolayer WS_2_ crystals would have the final configuration of the O_2_‐chemisorption rather than the O_2_‐dissociative chemisorption.^[^ ^14^ ^]^ The major molecules such as N_2_, O_2_, and H_2_O in air can be weakly physisorbed at both the pristine surface and defect sites of WS_2_. However, this physisorption has virtually no influence on the electronic and optical properties of the WS_2_ monolayer due to easy desorption of physisorbed molecules.^[^ ^14^ ^]^ In addition, our first‐principle calculations demonstrate that the oxygen molecules can only be chemisorbed onto the defects (sulfur vacancy) and attain a fully stable chemisorption state, indicating that the oxygen molecules can be majorly adsorbed onto the WS_2_ in the air. The detailed theoretical calculation results on molecular interactions with the sulfur vacancy and pristine surface of WS_2_ are provided in Sections S2 and S3 (Supporting Information). To investigate the role of oxygen fixation in the excitonic properties of monolayer WS_2_ with excluding the effects of disorders induced by the substrates, we studied *h*‐BN encapsulated WS_2_ crystals suspended on line trenches with a linewidth of 1.8 µm in comparison with bare WS_2_, as shown in Figure 1b,d. Scanning electron microscope images confirm the suspended structures for both the bare (Figure 1b) and *h*‐BN encapsulated (Figure 1d) WS_2_ crystals on the line trenches (see Figure S4, Supporting Information). The monolayered WS_2_ crystals used in this study were grown on sapphire substrates using a chemical vapor deposition (CVD) method.^[^ ^19^ ^]^ Mechanically exfoliated *h*‐BN flakes with a thickness of ≈40 nm were used as the encapsulating layers in the *h*‐BN/WS_2_/*h*‐BN structures. The detailed sample preparation processes are described in the methods. Figure 1c,e display the spatial photoluminescence profiles of the bare (Figure 1c) and *h*‐BN encapsulated (Figure 1e) WS_2_ suspended on the line trenches, respectively. The steady‐state photoluminescence measurements were carried out at a low level of excitation (≈0.065 kW cm^−2^) to rule out the heating effect. It is worth noting that the photoluminescence intensity becomes stronger in the suspended regions than in the supported regions for both the bare and *h*‐BN encapsulated WS_2_ crystals due to the enhanced local field effect by optical interference in the trench region.^[^ ^7^ ^]^ To confirm the exciton species, the photoluminescence spectra were measured at a cryogenic temperature of 77 K under a vacuum level of ≈1 × 10^−5^ Torr, as shown in Figure 1f. For the *h*‐BN encapsulated WS_2_, the neutral exciton (X^0^) and the trion (X^−^) are identified at energies of 2.042 and 2.001 eV, respectively, while the bare WS_2_ shows three species of the neutral exciton (X^0^), trion (X^−^), and defect‐related trapped exciton (L) at 2.087, 2.042, and 2.026 eV, respectively. The energy of neutral exciton was assigned by measuring the differential reflectance spectra (see Figure S5, Supporting Information), and those of trion and defect‐related trapped exciton were identified by the energy differences from the neutral exciton.^[^ ^20^, ^21^ ^]^ Compared to the bare WS_2_ showing the emission prevailed by the trion and the defect‐related trapped exciton, the *h*‐BN encapsulated WS_2_ exhibits the predominant emission from the neutral exciton with homogeneous linewidth of the 6 meV,^[^ ^9^ ^]^ indicating that the defect‐induced free electrons and inhomogeneous broadening are substantially reduced by the encapsulating WS_2_ with *h*‐BN layers. These results indicate that the oxygen fixation by *h*‐BN encapsulation can play a crucial role in the defect removal with reducing the free electron density. To directly confirm the fixation effect of the adsorbed oxygen molecules on the defects, the change in excitonic spectra was monitored under the different ambient conditions of air and vacuum, as shown in Figure 1g (see Figure S6, Supporting Information). Striking features are observed for the bare WS_2_ crystals, showing that the spectral weight of neutral excitons is predominant over that of trions under ambient air condition, whereas that of trions becomes larger than that of neutral excitons under vacuum. These results indicate that the oxygen adsorbates on the defect sites are released by changing the ambient condition from air to vacuum, raising the density of free electrons in the bare WS_2_ crystals under vacuum.^[^ ^16^, ^17^ ^]^ As shown in Figure S7 (Supporting Information), the *h*‐BN encapsulated WS_2_ samples fabricated under an inert (N_2_) environment exhibit much stronger trion intensity (higher free electron concentration) compared to that of the *h*‐BN encapsulated WS_2_ fabricated in the air. The spectral feature is very similar to that of the bare WS_2_ measured in the vacuum environment (see top panels in Figure 1f,g). Furthermore, the exfoliated monolayer WS_2_ and WSe_2_ with a lower density of chalcogen vacancies give rise to a less change in the free electron density against the variation of the ambient conditions, implying that the oxygen molecules are mostly adsorbed on the chalcogen vacancies (see Figure S8, Supporting Information). In contrast, the *h*‐BN encapsulated WS_2_ crystals exhibit almost the same spectra prevailed by the neutral excitons regardless of the ambient conditions, highlighting that the *h*‐BN encapsulation effectively removes the internal defects by stabilizing the oxygen molecules adsorbed onto WS_2_ crystals.

**Figure 2:**
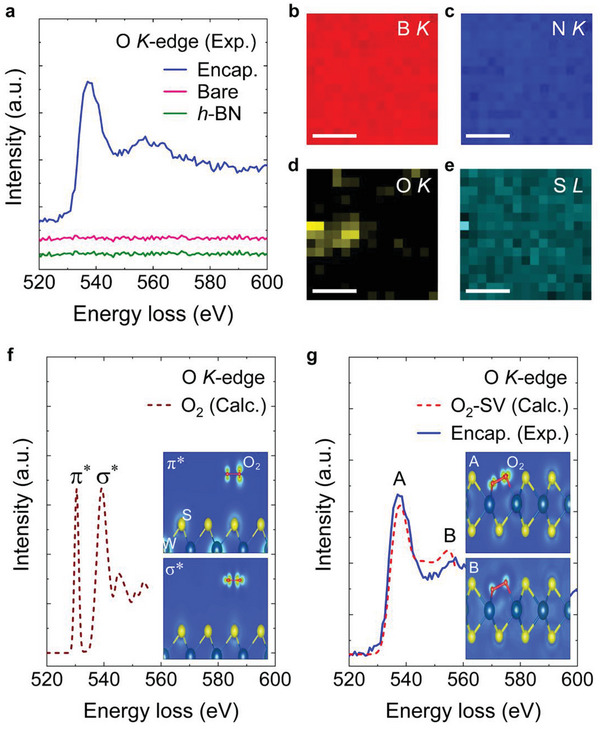
a) Experimental oxygen *K*‐edge EELS spectra for the *h*‐BN encapsulated WS_2_, bare WS_2_, and *h*‐BN flake crystals. b–e) The EELS maps for the boron (b), nitrogen (c), oxygen (d) *K*‐edge, and the sulfur (e) *L*‐edge measured from *h*‐BN encapsulated WS_2_. The scale bars are 100 nm. f) The calculated oxygen *K*‐edge EELS spectrum ε_2_(ω) for a physisorbed oxygen molecule on the pristine WS_2_. g) The calculated oxygen *K*‐edge EELS spectrum ε_2_(ω) (red dashed) for a chemisorbed oxygen molecule on the sulfur vacancy (O_2_‐SV) of the WS_2_. The experimental oxygen *K*‐edge spectrum (blue line) for the *h*‐BN encapsulated WS_2_ is in good agreement with the calculation result (red dashed). The insets of (f,g) represent the real‐space orbital distributions ρ(***r***)_*E*_ of the π* (*E* ≈ 530 eV) and σ* (*E* ≈ 539 eV) for (f) and the A (*E* ≈ 538 eV) and B (*E* ≈ 555 eV) for (g), respectively.

The EELS analysis for oxygen *K*‐edge confirms that the *h*‐BN encapsulation anchors the chemisorbed oxygen molecules onto the defects of monolayer WS_2_ crystals. **Figure** **2**a displays the EELS spectra of the oxygen *K*‐edge for the *h*‐BN encapsulated WS_2_, bare WS_2_, and *h*‐BN flake crystals. The *h*‐BN encapsulated WS_2_ crystals show the oxygen *K*‐edge peaks centered at 538 and 556 eV, whereas any oxygen‐related features are not observed for the bare WS_2_ and the *h*‐BN crystals. This suggests that the adsorbed oxygen molecules on WS_2_ are fixated by the *h*‐BN encapsulation (see Figure S9, Supporting Information). Figure 2b–e shows the EELS maps for the boron (b), nitrogen (c), oxygen (d) *K*‐edge, and the sulfur (e) *L*‐edge measured from the *h*‐BN encapsulated WS_2_. Note that the elemental signal displayed for a pixel in the EELS map is the sum of the elemental signals detected through 2D scanning using an electron beam with a spatial resolution of 1 Å over an area of 20 nm × 20 nm. The spatial EELS maps confirm that the oxygen *K*‐edge signal is markedly weak compared to those of other elements, and is randomly distributed in the 2D plane of *h*‐BN encapsulated WS_2_ samples. This strongly indicates that the oxygen molecules are chemisorbed on the randomly distributed local defects in WS_2_ crystals (see Figure S10, Supporting Information).

To discover the adsorption types of the adsorbed oxygen molecules by *h*‐BN encapsulation, we demonstrate the theoretical EELS spectra of oxygen *K*‐edge for the physisorbed oxygen molecule on the pristine WS_2_ (Figure 2f) and chemisorbed oxygen molecule at the sulfur vacancy site of the WS_2_ (Figure 2g). To understand the underlying physics for the EELS results, we performed theoretical EELS calculations based on the first‐principles calculations implemented in the full‐potential linearized plane wave (FLAPW) + local orbitals with the ELK code. Here, we used the pseudo core‐hole method, where the self‐consistent calculation is made in terms of one of the oxygen nuclei constrained to be positively charged (+1e) and an additional electron (−1e) is simultaneously constrained to occupy the conduction orbitals. After the self‐consistent calculation, the Kohn‐Sham orbitals become well defined, and then we compute the photon‐absorption matrix elements between the core orbital (*s*‐orbital) of the oxygen nuclei and the unoccupied conduction bands, which corresponds to the dielectric function for EELS spectra (see Section S11, Supporting Information for more detail).

The calculated EELS spectrum for the physisorbed oxygen molecule on the pristine WS_2_ indicates the two main peaks at 530 and 539 eV, which originate from the transition of the core electrons to two kinds of trivial hybridized states in the oxygen molecule, featured as anti‐bonding orbital π* (top) and σ* (bottom) distributions, respectively (see the inset of Figure 2f), whereas the calculated oxygen *K*‐edge peaks for the chemisorbed oxygen molecule appear at the energy loss positions of 538 and 555 eV (labelled as A and B on the spectrum), respectively. Since the core‐hole excitation makes the oxygen molecule to have an asymmetric potential, the π* orbital distribution in the inset reflects asymmetric densities. As shown in Figure 2g, the calculated EELS spectrum (red dashed) for a chemisorbed oxygen molecule is in good agreement with that of the *h*‐BN encapsulated WS_2_ (blue line), indicating that the fixated oxygen molecules by the *h*‐BN encapsulation are chemisorbed at the defect sites of the WS_2_. The anti‐bonding orbital π* peak (530 eV) is absent for the chemisorbed oxygen molecule in the calculated EELS spectrum. The disappearance of π‐bonding is commonly interpreted as a transformation of bonding sequences.^[^ ^22^ ^]^ In our study, it is found that the hybridizations between the oxygen molecule and the surrounding tungsten atoms directly suppress the π* peak. As shown in the top inset of Figure 2g, the real‐space orbital distribution for the peak A resembles an orbital shape of the σ* peak for the physisorbed oxygen molecule, indicating that the hybridizations between the oxygen molecule and the tungsten atoms induce σ* antibonding energy state similar to that of the physisorbed oxygen molecule. In contrast, for the peak B, the real‐space orbitals show highly delocalized distribution for both the oxygen molecule and the WS_2_ crystal (the bottom inset of Figure 2g), implying that the peak B is due to the transition of the core electrons to continuum bands contributed from both the oxygen molecules and the WS_2_ crystals.

**Figure 3:**
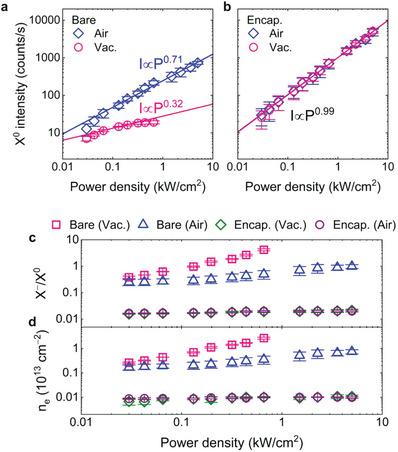
a,b) Neutral exciton (X^0^) emission intensity of the bare (a) and *h*‐BN encapsulated (b) WS_2_ crystals as a function of the excitation power density at room temperature. The blue diamond and pink circle symbols indicate the power‐dependent exciton emission intensity measured under ambient air and vacuum conditions. c) Photoluminescence intensity ratio (X^−^/X^0^) of the trion to the neutral exciton as a function of the excitation power density. d) Free electron density as a function of the excitation power density by using the mass action law. The pink square (blue triangle) and olive diamond (purple circle) symbols represent the X^−^/X^0^ and free electron density of the bare and *h*‐BN encapsulated WS_2_ under vacuum (air) ambient conditions, respectively. The error bars in (a−d) indicate the standard deviation of the measured data.

The investigation of the exciton recombination processes against the excitation power for the bare and *h*‐BN encapsulated WS_2_ crystals reveals that the oxygen fixation suppresses the nonradiative decay for the neutral excitons by stabilizing the free electron density of the WS_2_. **Figure** **3**a,b shows the double‐logarithmic plots of the neutral exciton (X^0^) emission intensity of the bare and *h*‐BN encapsulated WS_2_ crystals as a function of the excitation power density under ambient air and vacuum conditions at room temperature, respectively. The bare WS_2_ crystal in air exhibits a sublinear increase with an exponent of 0.71 in a power‐law (*I ∝ P^α^*, *P*: excitation power), while that in vacuum shows a smaller exponent of 0.32. The power dependence can be understood by introducing a simple rate equation model for the steady‐state photoluminescence. The rate equation for exciton generation and recombination can be described by^[^ ^23^, ^24^ ^]^

(1) $$G\;=\;\frac{n_{X}}{\tau_{X}}\;+\;Tn_{X}n_{e}\;+\;An_{X}n_{e}\;+\;\gamma n_{X}^{2}$$

where *n_X_* is the neutral exciton density, τ_*X*_ is the exciton lifetime, *n_e_* is the electron density, *T* is the trion formation coefficient, *A* is the exciton‐electron Auger coefficient, and γ is the exciton–exciton annihilation coefficient. At our excitation power range, the exciton density is estimated to be from the low 10^10^ to the low 10^11^ cm^−2^. In this range of exciton density, it has been known that the exciton‐exciton annihilation process becomes negligible.^[^ ^25^ ^]^ Furthermore, the recent work has shown that the photogenerated excitons are mostly converted to the trions in this range, indicating that the Auger process can also be neglected.^[^ ^24^ ^]^ Thus, the predominant nonradiative decay channel of the neutral excitons is the exciton‐to‐trion conversion process (see Figure S13, Supporting Information). When the free electron density increases with the power dependence of *P^μ^*, the neutral excitons are converted to the trions in proportional to *P^μ^*, and the neutral exciton emission intensity follows the power‐law of *P^1−μ^* (see Section S12, Supporting Information). The sublinear increase in the exciton emission in bare WS_2_ indicates that the free electron density increases with increasing excitation power density by releasing oxygen molecules chemisorbed on defect sites, promoting the trion conversion process. The smaller exponent further confirms the easier desorption under ambient vacuum condition. Interestingly, the *h*‐BN encapsulated WS_2_ shows a linear increase with an exponent of 0.99 for both the air and vacuum ambient conditions, as shown in Figure 3b. This indicates that *h*‐BN encapsulation results in predominant neutral exciton emission over the decay channels without generating free electrons by desorption.^[^ ^16^, ^24^ ^]^ Figure 3c shows the photoluminescence intensity ratio (X^−^/X^0^) of the trion to the neutral exciton as a function of the excitation power density. The X^−^/X^0^ of bare WS_2_ measured under ambient vacuum condition steeply increases from 0.38 to 4.18, while the X^−^/X^0^ under the air environment gradually increases from 0.25 to 0.50 over the excitation power density range from 0.03 to 0.66 kW cm^−2^. These further evidences that the desorption process, which induces free electrons and exciton‐to‐trion conversion, is much facilitated in vacuum than in air. In striking contrast, the X^−^/X^0^ of *h*‐BN encapsulated WS_2_ is kept almost constant at ≈0.02 over the excitation power densities under both the ambient air and vacuum conditions, which is greatly reduced by more than one order of magnitude compared to that of the bare WS_2_. This clearly indicates that *h*‐BN encapsulation prevents the desorption of oxygen molecules by the laser illumination, resulting in predominant recombination by neutral excitons. By considering the intensity weight ($I_{X^{-}}/I_{total}$) of the trion in the photoluminescence spectra, we estimated the free electron density as a function of the excitation power density by using the mass action law (see Section S14, Supporting Information).^[^ ^26^, ^27^ ^]^ As shown in Figure 3d, the free electron density exhibits a very similar trend as X^−^/X^0^. For bare WS_2_, the free electron density drastically increases and reaches 2.66 × 10^13^ cm^−2^ at an excitation power density of 0.66 kW cm^−2^ in vacuum, which is close to the Mott density (≈1 × 10^14^ cm^−2^),^[^ ^28^ ^]^ while gradually increasing to 7.38 × 10^12^ cm^−2^ at an excitation power density of 4.99 kW cm^−2^ in air. However, regardless of the ambient conditions, the free electron density of *h*‐BN encapsulated WS_2_ is maintained at ≈9 × 10^10^ cm^−2^ with increasing excitation power, resulting from the fixation of chemisorbed oxygen molecules by *h*‐BN layers. Additonally, using the bare and *h*‐BN encapsulated WS_2_ capacitor devices, we estimated the free electron densities in the bare and *h*‐BN encapsulated WS_2_ at the vacuum and air environments. For the bare WS_2_, the electron densities were estimated to be 1.15 × 10^13^ cm^−2^ (vacuum) and 3.69 × 10^12^ cm^−2^ (air) at *V_g_* ≅0 *V* (gate voltage), respectively, while those of *h*‐BN encapsulated WS_2_ were estimated to be 2.05 × 10^11^ cm^−2^ at both the vaccum and air environments. These electron densities were in good agreement with those estimated using the mass‐action law. From the difference in free electron densities in‐between the vacuum and air environments, we estimated the number of desorbed/adsorbed oxygen molecules on the sulfur vacanies to be 7.17 × 10^12^ cm^−2^ (see Section S15, Supporting Information).

**Figure 4:**
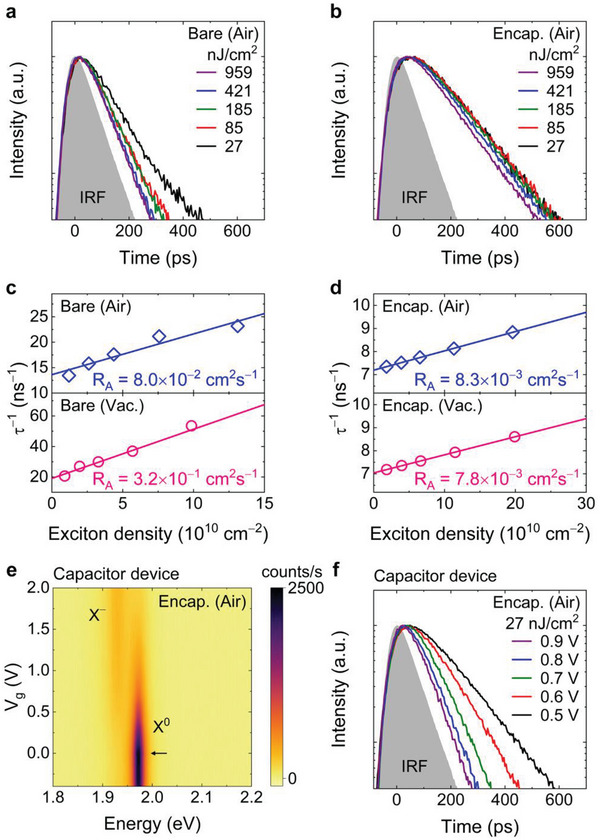
a,b) Photoluminescence decay curves of the neutral excitons measured as a function of the energy fluence for bare (a) and *h*‐BN encapsulated (b) WS_2_ under ambient air condition. c,d) Exciton density‐induced recombination rate (τ^−1^) for the bare (c) and *h*‐BN encapsulated (d) WS_2_ under ambient air and vacuum conditions, resulting in the exciton annihilation rate constant (*R_A_*) due to exciton‐to‐trion conversion process by a linear fit. e) Gate‐voltage‐dependent photoluminescence spectral map of the *h*‐BN encapsulated WS_2_ capacitor devices. The black arrow indicates the charge neutral point of the *h*‐BN encapsulated WS_2_ capacitor device. f) Gate‐voltage‐dependent photoluminescence decay curves of the neutral excitons in the *h*‐BN encapsulated WS_2_ capacitor device.

The nonradiative decay channel of neutral excitons can be attributed to exciton‐to‐trion conversion at our excitation power range. From the rate Equation (1), the total recombination rate (*R_T_*) can be described by  *R_T_* =  *R*_0_ + *R_A_n_X_*, where *R*_0_ is the density‐independent recombination rate and *R_A_* is the exciton annihilation rate constant due to the exciton‐to‐trion conversion process. The role of the oxygen fixation can be quantitatively understood by estimating the exciton annihilation rate constant (*R_A_*). To measure the exciton lifetime with increasing excitation power, we carried out time‐resolved photoluminescence spectroscopy. For bare WS_2_, the photoluminescence decay curves of neutral excitons under air ambient condition show a steep shortening of the decay time from 74 to 43 ps with increasing pump fluence from 27 to 959 nJ cm^−2^, as shown in **Figure** **4**a. However, the exciton decay curves for *h*‐BN encapsulated WS_2_ show a slight decrease in the lifetime above the pump fluence of 421 nJ cm^−2^, as shown in Figure 4b. It is noteworthy that the exciton lifetime in *h*‐BN encapsulated WS_2_ becomes longer than that of bare WS_2_, showing exciton lifetime of 136 ps in air at a pump fluence of 27 nJ cm^−2^. The decay spectra at vacuum are shown in Figure S16 (Supporting Information). The increase in exciton lifetime in the *h*‐BN encapsulated WS_2_ strongly suggests that the nonradiative decay by the trion formation, which occurs on a very fast time scale of a few ps, is significantly inhibited due to the passivation of defects by the oxygen fixation.^[^ ^23^, ^29^, ^30^ ^]^ To quantitatively evaluate the exciton annihilation rate constant (*R_A_*), the exciton density‐induced recombination rate (τ^−1^) was plotted as a function of the exciton density using the measured exciton lifetimes (see Section S17, Supporting Information).^[^ ^30^ ^]^ Note that the exciton density was estimated by calculating the net absorption in the monolayer WS_2_ for the pump fluence.^[^ ^31^, ^32^ ^]^ As presented in Figure 4c,d, the *h*‐BN encapsulated WS_2_ crystals exhibit an exciton annihilation rate constant of 8.3 × 10^−3^ cm^2 ^s^−1^ (7.8 × 10^−3^ cm^2^ s^−1^), while the bare WS_2_ crystals show a value of 8.0 × 10^−2^ cm^2^ s^−1^ (3.2 × 10^−1^ cm^2^ s^−1^) under air (vacuum) ambient conditions. As expected, the exciton annihilation rate constant of the *h*‐BN encapsulated WS_2_ is remarkably reduced by approximately two orders of magnitude compared to that of the bare WS_2_. These results are attributed to the suppression of exciton‐to‐trion conversion process in the *h*‐BN encapsulated WS_2_ due to the greatly lowered and stabilized free electron density. This fact can be additionally verified by investigating the decay dynamics of neutral excitons with an electrostatic doping in the *h*‐BN encapsulated WS_2_ capacitor devices at a fixted exciation power. Figure 4e shows the gate‐voltage‐dependent photoluminescence spectral map in the *h*‐BN encapsulated WS_2_ capacitor devices, showing that the charge neutral point is determined at *V_g_* ≅0 *V*. The increase in the gate voltage (*V_g_* > 0 *V*) gives rise to the gradual decrease in the emission intensity for the neutral exciton and, simultaneously, the increase in the emission intensity for the trion. This indicates that the increase in free electron density facilitates the exciton‐to‐trion conversion process, leading to the nonradiative decay of neutral excitons. The gate‐voltage‐dependent photoluminescence decay curves of neutral excitons also clearly show that the increase in the free electron density promotes the exciton annihiliation. As shown in Figure 4f, the decay time of neutral excitons in the *h*‐BN encapsulated WS_2_ capacitor devices becomes steeply shorten from 139 to 36 ps for increasing the gate voltage from 0.5 to 0.9 V. The exciton annihilation rate constant with the electrostatic doping is estimated to be 1.3 × 10^−1^ cm^2^ s^−1^ (see Section S18, Supporting Information), which is similar to that of bare WS_2_ under the vacuum ambient condition.

**Figure 5:**
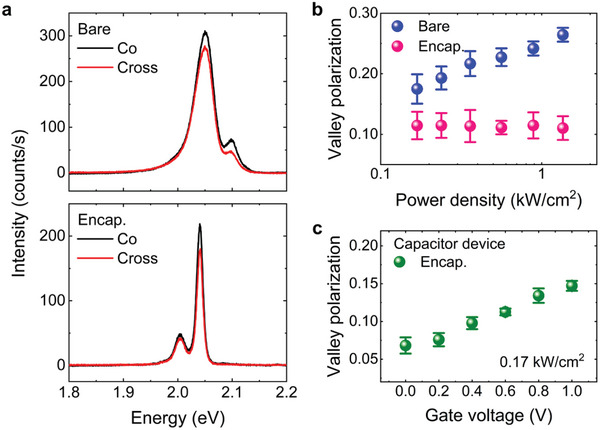
a) Circularly polarized photoluminescence spectra for the bare (top panel) and *h*‐BN encapsulated (bottom panel) WS_2_ measured at 77 K. b) Degree of valley polarization for the exciton emission in the bare and *h*‐BN encapsulated WS_2_ taken as a function of the excitation power density. c) Degree of valley polarization for the neutral excitons in the *h*‐BN encapsulated WS_2_ capacitor devices taken as a function of the gate voltage. The error bars marked in (b,c) exhibit the standard deviation of the measured valley polarization values.

The *h*‐BN encapsulation gives rise to an almost constant level of the free electron density in WS_2_ crystals for elevated excitation powers, which can provide a stable and robust valley polarization against various excitation conditions. In contrast, for bare WS_2_, the drastic increase in free electron density with increasing excitation would cause a large variation in the valley polarization due to the change in decay dynamics of the neutral excitons caused by the exciton‐to‐trion conversion.^[^ ^33^, ^34^ ^]^ To investigate the effect of *h*‐BN encapsulation on the valley polarization, we carried out circular polarization‐resolved photoluminescence measurements as a function of the excitation power density at 77 K. **Figure** **5**a show the circularly polarized photoluminescence spectra measured from the bare (top panel) and *h*‐BN encapsulated (bottom panel) WS_2_, respectively. As a result, the degree of valley polarization with increasing excitation power shows a very different trend for the bare and *h*‐BN encapsulated WS_2_ crystals, as shown in Figure 5b. An important distinction is that the *h*‐BN encapsulated WS_2_ exhibits a stable valley polarization ratio at a constant level, whereas that of the bare WS_2_ shows a large variation by changing the excitation power. The valley polarization can be described by the following equation *P_V_* = *P*_0_/(1 + 2τ_*X*_/τ_*V*_) ,^[^ ^35^ ^]^ where *P*_0_ is the initial valley polarization, τ_*X*_ is the valley exciton lifetime, and τ_*V*_ is the valley relaxation time. The initial valley polarization (*P*_0_) given by the optical selection rules can be assumed to be unity,^[^ ^5^ ^]^ meaning that the valley polarization (*P_V_*) is then mainly governed by the competition between the exciton lifetime and the valley relaxation time.^[^ ^35^ ^]^ As shown in Figure 5b, the degree of valley polarization of bare WS_2_ is 1.5 times higher than that of *h*‐BN encapsulated WS_2_ at the lowest excitation power density of 0.17 kW cm^−2^. This can be attributed to the fact that in bare WS_2_, the neutral excitons decay rapidly within the valley, rather than an intervalley scattering, due to a shortened exciton lifetime caused by a higher exciton‐to‐trion conversion rate. In addition, the large variation in the valley polarization of bare WS_2_ can be understood as a result of the change in the exciton lifetime by the accelerated trion conversion process with increasing excitation power, as shown in Figure 4a,c.^[^ ^33^, ^34^ ^]^ Accordingly, the valley polarization can be increased in the bare WS_2_ for elevated excitation powers. For the *h*‐BN encapsulated WS_2_, however, the exciton‐to‐trion conversion and the exciton lifetime are maintained at almost constant levels, resulting in a stable valley polarization ratio for elevated excitation powers. The large variation in the valley polarization is also observed in the *h*‐BN encapsulated WS_2_ capacitor devices with the electrostatic doping (Figure 5c), showing the drastic decrease in the exciton lifetime as the gate voltage increases (see Section S19, Supporting Information). This also confirms that the change in the free electron density leads to the large variation in the valley polarization.

## 3 Conclusion

In conclusion, we have demonstrated that *h*‐BN encapsulation greatly removes the defect‐related gap states by stabilizing the chemisorbed oxygen molecules onto the defects of monolayer WS_2_ crystals, that are provided during the interactions between WS_2_ and atmosphere. It is clearly shown that the oxygen molecules are chemisorbed onto the defects of WS_2_ crystals and are fixated by *h*‐BN encapsulation with excluding a possibility of oxygen molecules trapped in bubbles or wrinkles formed at the interface between WS_2_ and *h*‐BN, as confirmed by the EELS study. Optical spectroscopic studies show that *h*‐BN encapsulation prevents the desorption of oxygen molecules over various excitation and ambient conditions, resulting in a greatly lowered and stabilized free electron density in monolayer WS_2_ crystals. This suppresses the exciton annihilation processes by two orders of magnitude compared to that of bare WS_2_. Furthermore, due to the stabilized free electron density in the *h*‐BN encapsulated WS_2_ crystals, the valley polarization becomes robust against the various excitation and ambient conditions. Our findings provide insight into the role of *h*‐BN encapsulation and open up the possibility to control the defect states in 2D semiconductors through adsorbate‐engineered 2D heterostructures.

## 4 Experimental Section

### Sample Preparation

The monolayer WS_2_ crystals were grown on a sapphire substrate by chemical vapor deposition methods,^[^ ^19^ ^]^ and the *h*‐BN flakes with a thickness of ≈40 nm were prepared by mechanical exfoliations from bulk *h*‐BN single crystals. The *h*‐BN encapsulated WS_2_ structure was fabricated by sequential pick‐up processes using the dry van der Waals stacking method. Elvacite resin or polycarbonate (PC) was utilized as a polymer stamp for the pick‐up of layered materials. The assembled *h*‐BN/WS_2_/*h*‐BN structures and picked‐up WS_2_ crystals were released onto the line trenches by melting the polymer stamp, where the line trenches were fabricated through conventional photolithography and reactive ion etching processes using 400‐nm‐thick SiO_2_‐coated Si substrates. The polymer stamps were removed by immersing the fabricated sample in chloroform. Finally, both the *h*‐BN encapsulated and the bare WS_2_ onto line trenches were annealed at 350 °C to improve the coupling between the stacked layers and remove transfer residues.

### Optical Measurements

The steady‐state and time‐resolved photoluminescence measurements were performed using a home‐built confocal microphotoluminescence system. Using a 40× (0.6 NA) objective (Nikon), the excitation beam was focused, and the signal from the samples was collected through an optical fiber on the focal image plane. For the steady‐state photoluminescence measurements, an argon‐ion laser with a wavelength of 457.9 nm (continuous wave) was used as an excitation source. The photoluminescence spectra were resolved by a spectrometer (Acton SpectraPro 500i with 0.5 m focal length and 1200 grooves/mm grating) equipped with a charge‐coupled device (CCD) detector (Princeton Instruments, 512 × 2048 pixels). For the circular polarization measurements, a combination of linear polarizer and quarter waveplate was used to generate circularly polarized excitation light, while another pair of linear polarizer and quarter waveplate was set for a polarization analyzer before collecting the signal through the slit of the spectrometer. The degree of valley polarization is defined as ρ  =  [(*PL*(σ^+^) − *PL*(σ^−^)]/[*PL*(σ^+^) + *PL*(σ^−^)], where *PL*(σ^±^) are the photoluminescence intensity for σ^+^ and σ^−^ polarized light components under excitation with a σ^+^ or σ^−^ polarized laser beam. The time‐resolved photoluminescence measurements were carried out using a picosecond pulsed diode laser (PicoQuant, LDH‐P‐FA‐355) with a wavelength of 355 nm (FWHM = 56 ps) and repetition rate of 40 MHz. The exciton lifetimes were measured using a hybrid photomultiplier detector (PicoQuant, PMA hybrid series) and a time‐correlated single photon counting system (PicoQuant).

### Scanning Transmission Electron Microscopy (STEM) and EELS Analysis

STEM was used by Monochromated ARM‐200F (NEO‐ARM) in Korea Basic Science Institute (KBSI) operated at 200 kV. Gatan imaging filter (GIF) Continuum HR‐1066 spectrometer was used to collect electron energy loss spectra

## Conflict of Interest

The authors declare no conflict of interest.

## Supporting information

Supporting Information

## Data Availability

The data that support the findings of this study are available from the corresponding author upon reasonable request.
