# Supplementary material for: Defect Passivation of 2D Semiconductors by Fixating Chemisorbed Oxygen Molecules via h‐BN Encapsulations
Source: Adv Sci (Weinh). 2024 Mar 17;11(22):2310197. doi: 10.1002/advs.202310197 (PMC11165525; doi:10.1002/advs.202310197)
Supplement: Supplementary file 1 — Supporting Information [file ADVS-11-2310197-s001.pdf]

ADVANCED SCIENCE
Open Access

Supporting Information

for *Adv. Sci.*, DOI 10.1002/advs.202310197

Defect Passivation of 2D Semiconductors by Fixating Chemisorbed Oxygen Molecules via *h*-BN Encapsulations

*Jin-Woo Jung, Hyeon-Seo Choi, Young-Jun Lee, Youngjae Kim, Takashi Taniguchi, Kenji Watanabe, Min-Yeong Choi, Jae Hyuck Jang, Hee-Suk Chung, Dohun Kim, Youngwook Kim and Chang-Hee Cho**

Supporting Information

Defect Passivation of Two-Dimensional Semiconductors by Fixating Chemisorbed Oxygen Molecules via *h*-BN Encapsulations

*Jin-Woo Jung, Hyeon-Seo Choi, Young-Jun Lee, Youngjae Kim, Takashi Taniguchi, Kenji Watanabe, Min-Yeong Choi, Jae Hyuck Jang, Hee-Suk Chung, Dohun Kim, Youngwook Kim, and Chang-Hee Cho**

Jin-Woo Jung, Hyeon-Seo Choi, Young-Jun Lee, Dohun Kim, Youngwook Kim, Chang-Hee Cho
Department of Physics and Chemistry, Daegu Gyeongbuk Institute of Science and Technology (DGIST), Daegu 42988, South Korea
E-mail: chcho@dgist.ac.kr

Youngjae Kim
School of Physics, Korea Institute for Advanced Study (KIAS), Seoul 02455, South Korea

Takashi Taniguchi
International Center for Materials Nanoarchitectonics, National Institute for Materials Science, Tsukuba 305-0044, Japan

Kenji Watanabe
Research Center for Functional Materials, National Institute for Materials Science, Tsukuba 305-0044, Japan

Min-Yeong Choi, Jae Hyuck Jang, Hee-Suk Chung
Electron Microscopy and Spectroscopy Team, Korea Basic Science Institute, Daejeon 34133, South Korea

Jae Hyuck Jang
Graduate School of Analytic Science and Technology, Chungnam National University, Daejeon 34134, South Korea

S1. Kinetic barriers for O_2-chemisorption and O_2-dissociative chemisorption processes

We performed density functional theory calculations implemented in the quantum espresso code, employing Optimized Norm-Conserving Vanderbilt (ONCV) pseudopotentials and the nudged elastic band (NEB) methods for 3 by 3 superstructures of monolayer WS_2. These calculations, based on the grimme-D2 van der Waals corrections, reveal the relative energy evolution for each reaction path from the initial configurations to the final configurations.

Figures S1a and S1b show the kinetic barriers for the O_2-chemisorption and O_2-dissociative chemisorption processes in the monolayer WS_2 (a) and WTe_2 (b) with the sulfur vacancy (SV). In the case of the WS_2, the kinetic barrier for the O_2-chemisorption (0.56 eV) is lower than that for the O_2-dissociative chemisorption process (0.76 eV), as shown in Figure S1a. From the transition state theory, the reaction rate is given by $k \cong f\exp(-E_b/k_BT)$, where k is the reaction rate, f is the attempt frequency, E_b is the barrier, k_B is the Bolzmann constant, and T is the temperature. The attempt frequency can be approximated by the value of 10^{12} s^{-1}.[1] The reaction rate (T = 300 K) for the O_2-chemisorption and dissociative chemisorption processes is estimated to be approximately 180 s^{-1} (E_b = 0.56 eV) and 0.17 s^{-1} (E_b = 0.76 eV), respectively. This result indicates that the probability of the O_2-chemisorption is 1000 times higher than that of the O_2-dissociative chemisorption. Thus, the oxygen chemisorption on the monolayer WS_2 crystals would have the final configuration of the O_2-chemisorption rather than the O_2-dissociative chemisorption.[2]

On the other hand, in the case of the WTe_2 (Figure S1b), there are no kinetic barriers (0.00 eV) for both the O_2-chemisorption and O_2-dissociative chemisorption processes, indicating that the oxygen chemisorption spontaneously occurs toward the O_2-dissociative chemisorption process in the case of WTe_2. These results are in good agreement with previous theoretical studies, showing that the type of the oxygen chemisorption depends on TMD materials.[2]

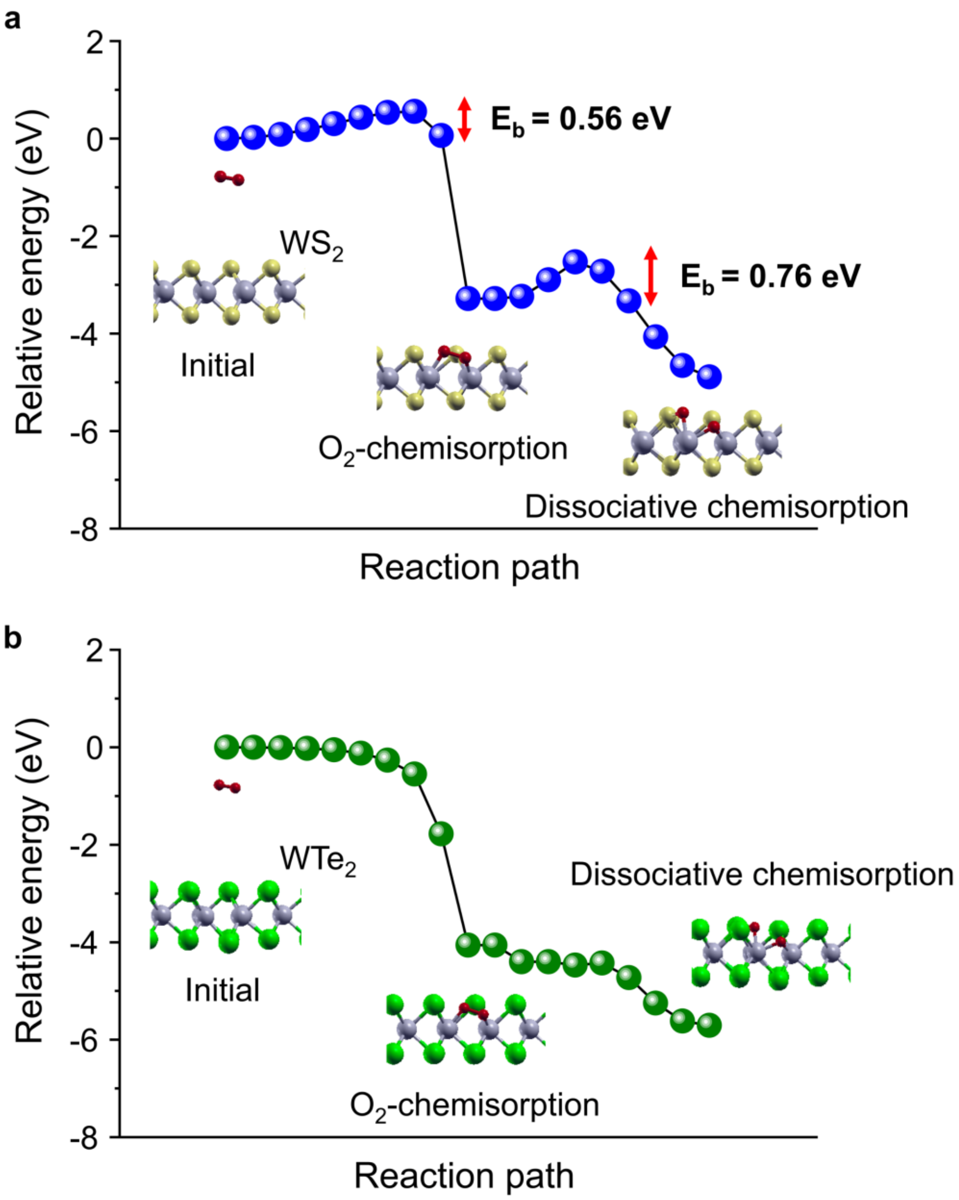

Figure S1. (a,b) Calculated reaction path and kinetic barriers for the O_2-chemisorption and O_2-dissociative chemisorption processes in the monolayer WS_2 (a) and WTe_2 (b) with the sulfur vacancy, respectively.

S2. Molecular interactions with the pristine surface and sulfur vacancy of WS_2

Figures S2 and S3 reveal the relative energy evolutions as a function of the reaction path for N_2, O_2, and H_2O with both the pristine WS_2 and SV of WS_2. In this reaction path, the decrease in relative energy indicates that the molecular adsorption proceeds toward a physisorption, corresponding to an exothermic process.[3] Meanwhile, an increase in relative energy exhibits that the molecular adsorption proceeds toward a chemisorption, which leads to an activation barrier in the chemical bonding sequence.

As shown in Figure S2, the N_2 molecule does not achieve a structurally favorable state when chemisorbed onto the SV. However, the N_2 exhibits a preference for physisorption (−0.08 eV) just prior to forming a chemical bond with surrounding tungsten atoms. Conversely, the O_2 starting from a weakly physisorbed state displays a minor repulsive barrier of ~0.56 eV just before chemically bonding. Subsequently, it attains a fully stable chemisorption state with the SV site, resulting a favorable energy of −3.28 eV. On the other hand, the H_2O prefers a physisorption-like configuration with the SV of WS_2, which does not show a chemisorption configuration.

Figure S3 shows the first-principles NEB calculation results for the molecular interaction with pristine WS_2. In case of the physisorption, all three molecules can be weakly physisorbed with the pristine WS_2. However, the weak adsorption energies in the final states lead to unstable physisorption configurations, resulting in the easy desorption of physisorbed molecules on pristine WS_2 surface. Thus the physisorption has virtually no influence on the electronic and optical properties of the WS_2 monolayer.

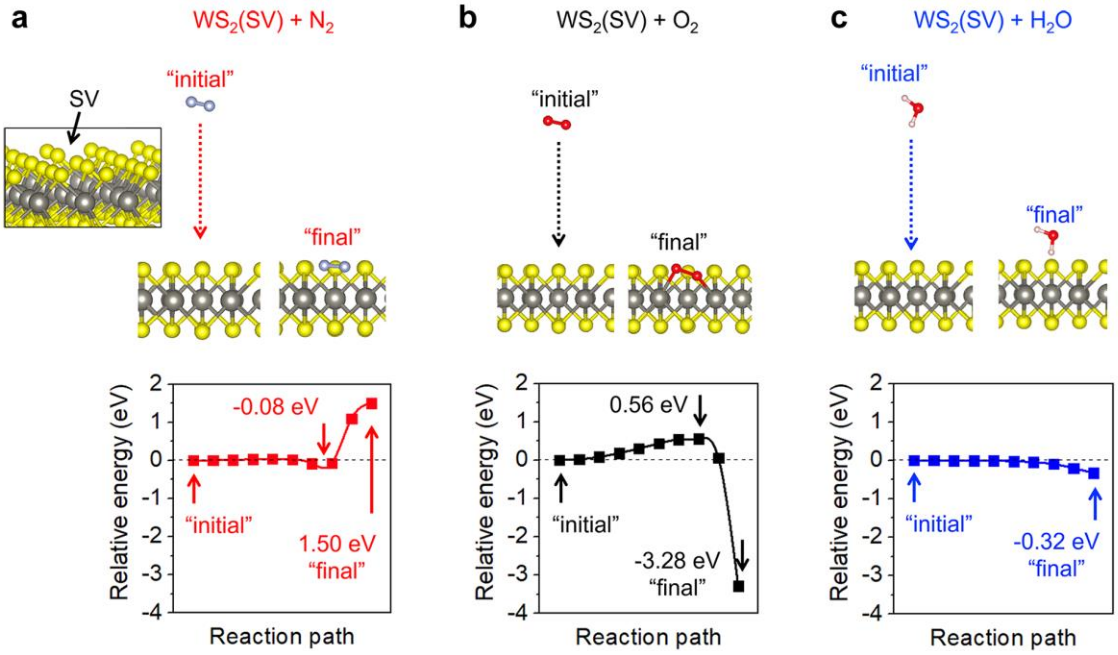

Figure S2. First-principles NEB calculations for molecular interactions with SV of WS_2. (a–c) The relative energy evolution of the N_2 (a), O_2 (b), and H_2O (c) for each reaction path from the initial configurations to the final configurations. The relative energy is defined as the total energy difference compared to that of the initial. Adsorption of a molecule at given surface has two different forms, physisorption and chemisorption. The physisorption reflects an exothermic process, resulting in a decrease in relative energy. The chemisorption, on the other hand,

exhibits an activation barrier in the chemical bonding sequence, leading to an increase in relative energy. (a) The N_2 molecule does not achieve a structurally favorable chemisorption state when adsorbed onto the SV. (b) The O_2 molecule can be only chemisorbed onto the SV and attain a fully stable chemisorption state (−3.28 eV). (c) The H_2O molecule prefers a physisorption-like configuration with the SV of WS_2, which does not show a chemisorption.

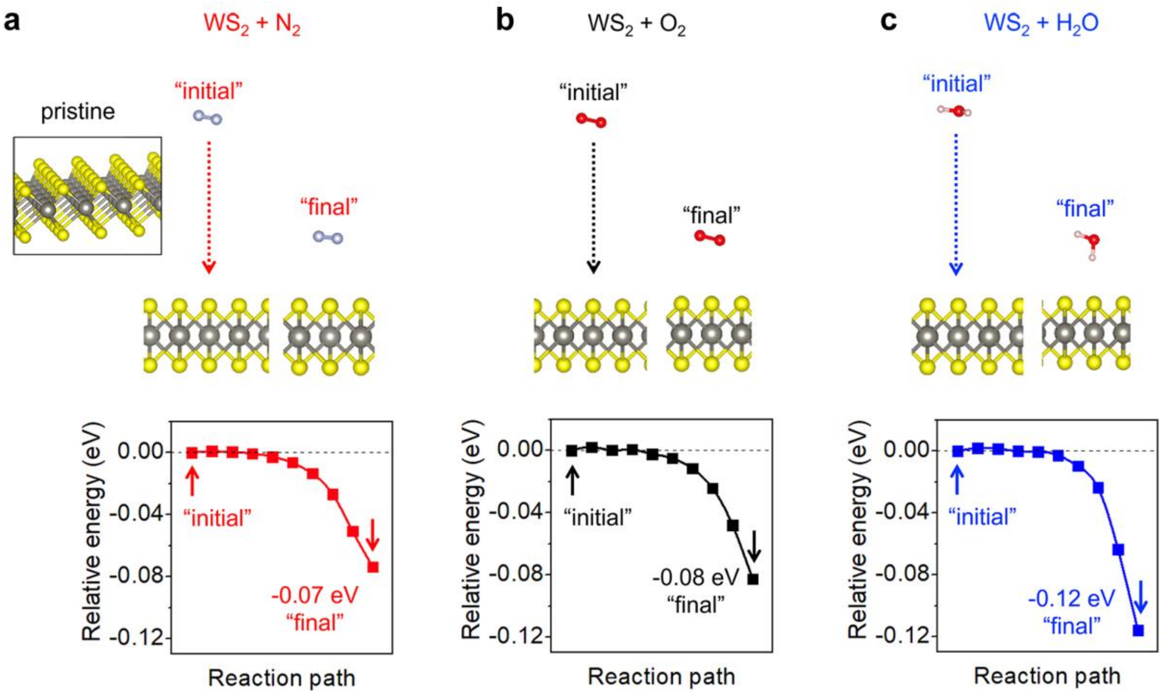

Figure S3. First-principles NEB calculations for molecular interactions with pristine WS_2. (a−c) The relative energy evolution of the N_2 (a), O_2 (b), and H_2O (c) for each reaction path from the initial configurations to the final configurations.

S4. Bare and *h*-BN encapsulated WS_2 suspended on the line trenches

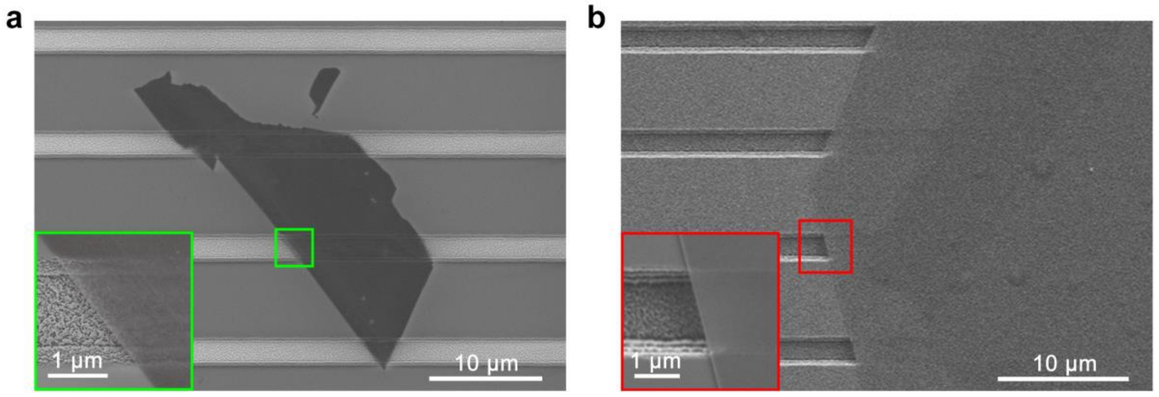

Figure S4. a,b) Scanning electron microscope images of the bare and *h*-BN encapsulated WS_2 crystals on the line trenches. The insets of (a) and (b) are the zoom-in images for the square marks of green and red boxes.

S5. Exciton species in the bare and *h*-BN encapsulated WS_2

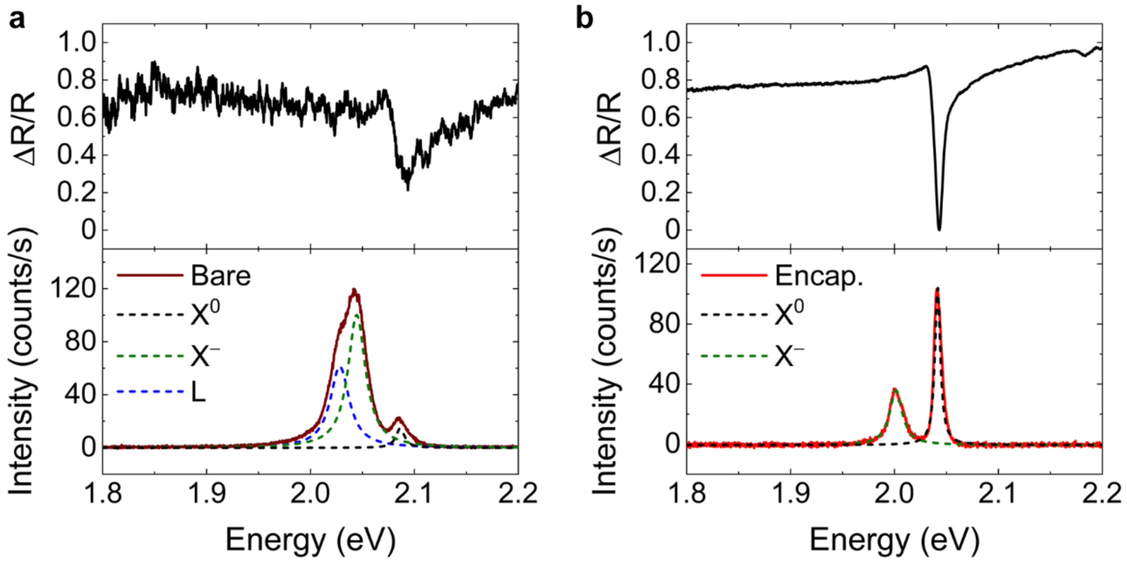

Figure S5. a,b) Differential reflectance and photoluminescence spectra measured from the bare (a) and *h*-BN encapsulated (b) WS_2 at the cryogenic temperature of 77 K. From the differential reflectance measurements, the exciton energy for the bare and *h*-BN encapsulated WS_2 was assigned to be 2.087 and 2.042 eV, respectively. By considering the peak separation between the exciton species based on the literatures,[4,5] the photoluminescence spectrum for the bare WS_2 is deconvoluted to three excitonic species corresponding to the neutral exciton (X^0), trion (X^-), and defect-related trapped exciton (L) states at 2.087, 2.042, and 2.026 eV, respectively. For the *h*-BN encapsulated WS_2, the two exciton species are assigned to be the neutral exciton (X^0), trion (X^-) at 2.042 and 2.001 eV, respectively. Note that the redshift of the exciton and trion energy in the *h*-BN encapsulated WS_2 compared with the bare WS_2 is due to the increase in the dielectric constant of the environment by the *h*-BN encapsulation.[6]

S6. Excitonic spectra of the bare and *h*-BN encapsulated WS_2 under the different ambient conditions

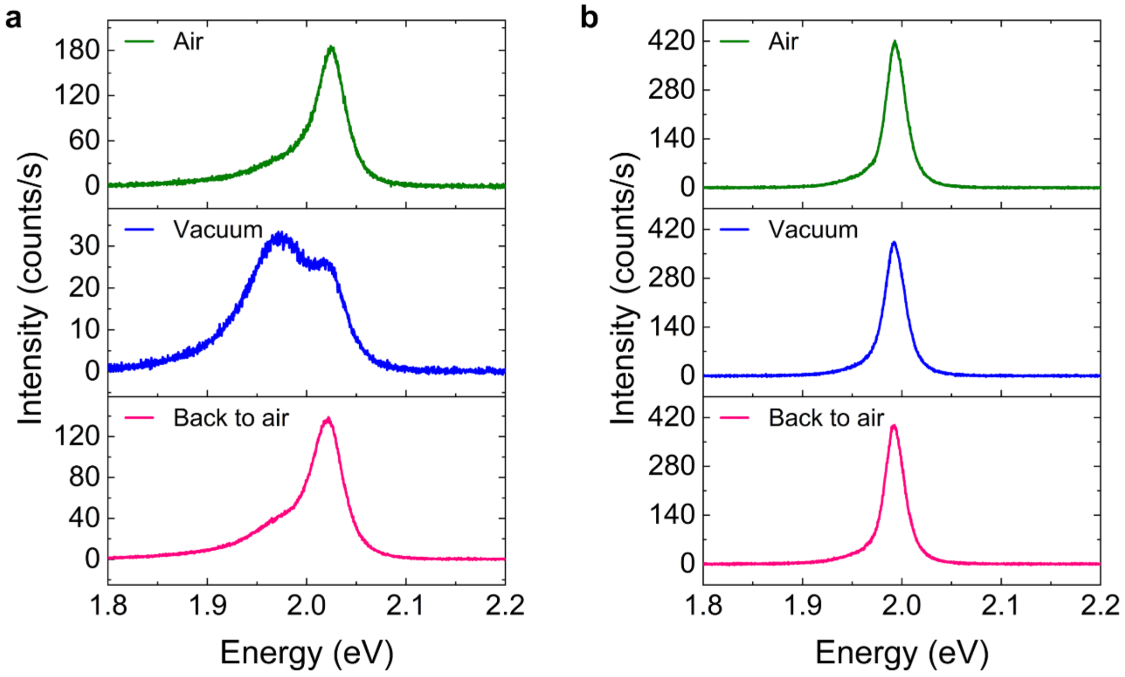

Figure S6. a,b) Photoluminescence spectra for the bare (a) and *h*-BN encapsulated (b) WS_2 measured under the ambient conditions of air, vacuum, and then air again. The photoluminescence spectral features of bare WS_2 are significantly altered according to the change in ambient condition, while those of *h*-BN encapsulated WS_2 are kept almost constant regardless of the change in ambient condition.

S7. *h*-BN encapsulated WS_2 fabricated under an inert gas environment

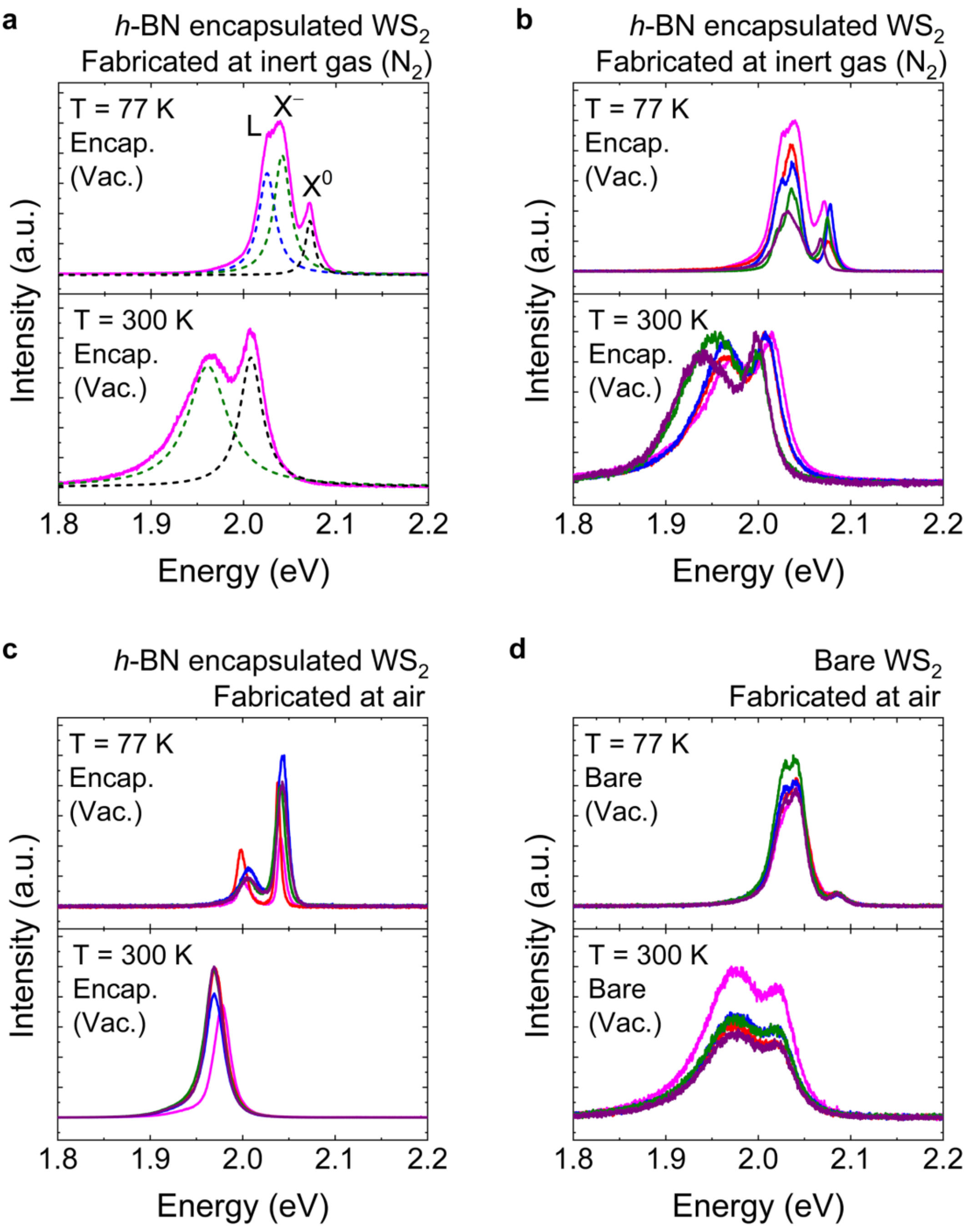

Figure S7. a) Photoluminescence spectra measured at the temperature of 77 K (top panel) and 300 K (bottom panel) for the *h*-BN encapsulated WS_2 fabricated under an inert environment. The black, olive, and blue dashed lines represent the neutral exciton (X^0), trion (X^-), and defect-related trapped exciton (L) states, respectively. b-d) Five representative spectra measured over many *h*-BN encapsulated WS_2 samples fabricated under the inert (b) and air (c) environments as well as the bare WS_2 (d) fabricated under the air environment.

S8. Spectral change in the exfoliated monolayer WS_2 and WSe_2 with a lower density of chalcogen vacancies by varying the ambient conditions

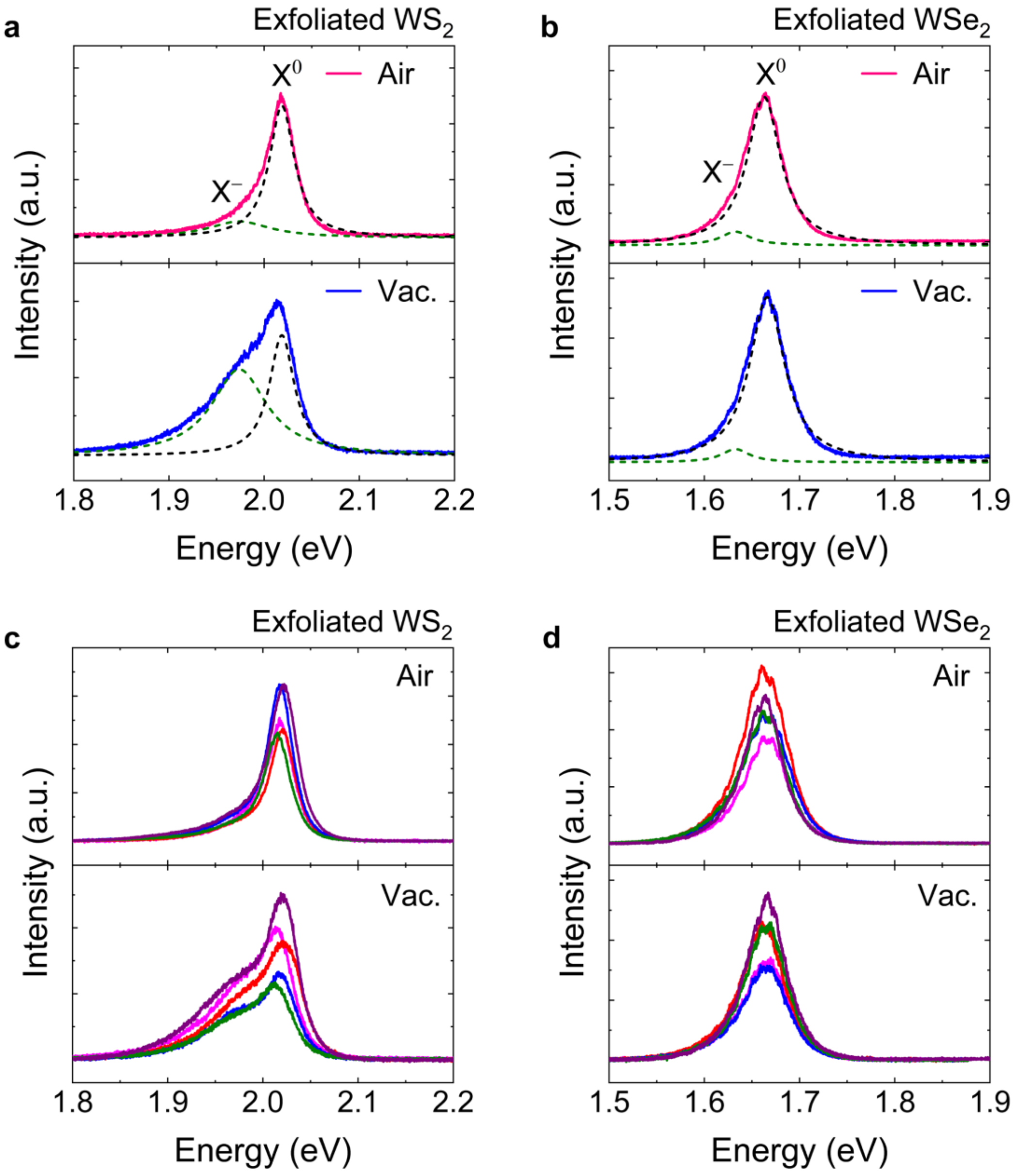

Figure S8. a,b) Photoluminescence spectra measured under the air (top panel) and vacuum (bottom panel) ambient conditions for the exfoliated monolayer WS_2 (a) and WSe_2 (b). The black and olive dashed lines represent the neutral exciton (X^0) and trion (X^-), respectively. c,d) Five representative spectra showing almost the same features over many WS_2 (c) and WSe_2 (d) samples under the variation of the air (top panel) and vacuum (bottom panel) ambient conditions.

S9. Electron energy loss spectroscopy spectra

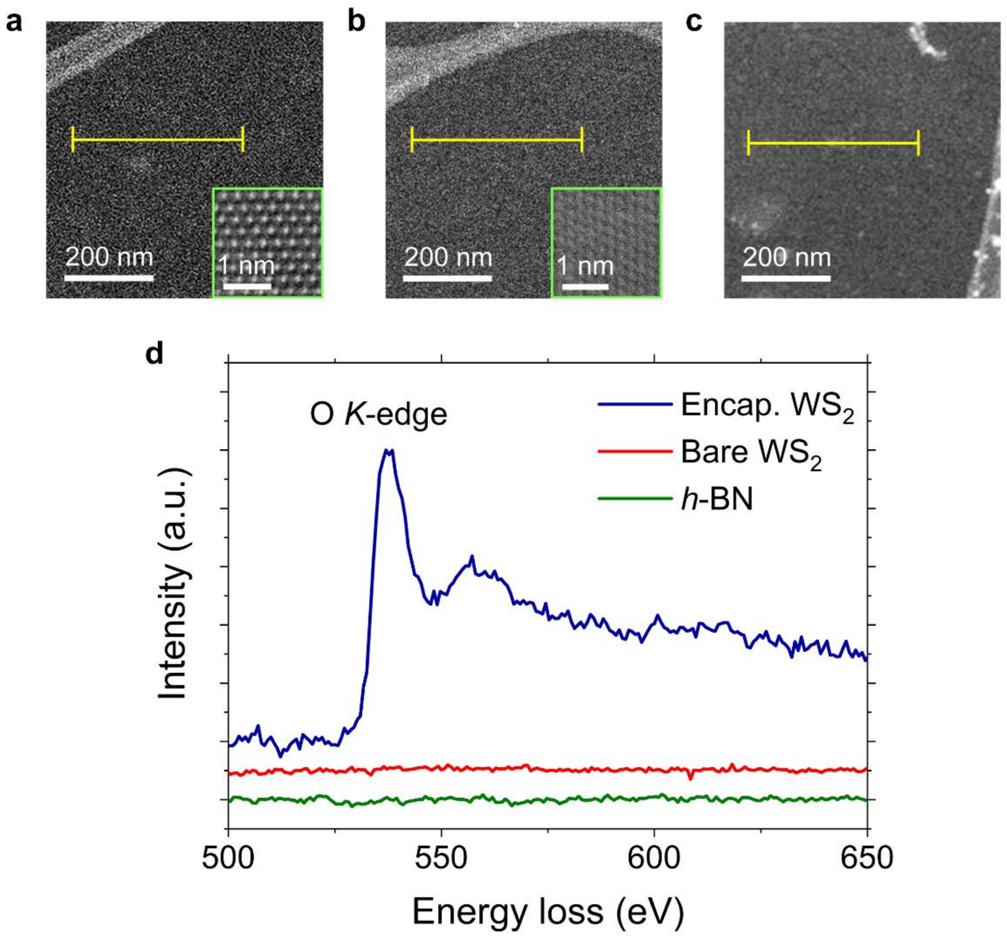

Figure S9. a–c) Annular dark field (ADF)-scanning transmission electron microscopy (STEM) images for bare WS_2 (a), *h*-BN flake (b), and *h*-BN encapsulated WS_2 crystals (c). Insets of (a,b) present the atomic-resolution ADF-STEM images for the bare WS_2 and *h*-BN flake. d) Oxygen *K*-edge electron energy loss spectroscopy (EELS) spectra for the *h*-BN encapsulated WS_2, bare WS_2, and *h*-BN flake. The EELS spectra are obtained from line-scanning for the regions (0.4 μm) marked as yellow bars in (a–c), and are acquired for a dwell time of 0.04 s per pixel. Note that in the case of the *h*-BN encapsulated WS_2, the strong boron and nitrogen signals in a few-nanometer-thick top and bottom *h*-BN layers encapsulating monolayer WS_2 interfere with the ability to obtain clear atomic-resolution STEM images of the monolayer WS_2. This makes it very challenging to clearly visualize the WS_2 plane in STEM images for *h*-BN encapsulated WS_2. More importantly, directly observing light atoms such as oxygen is challenging due to their easy knockout under the electron beam irradiation as well as the very weak TEM contrast, as widely reported in the previous works.[7,8]

S10. Electron energy loss spectroscopy maps for *h*-BN encapsulated WS_2

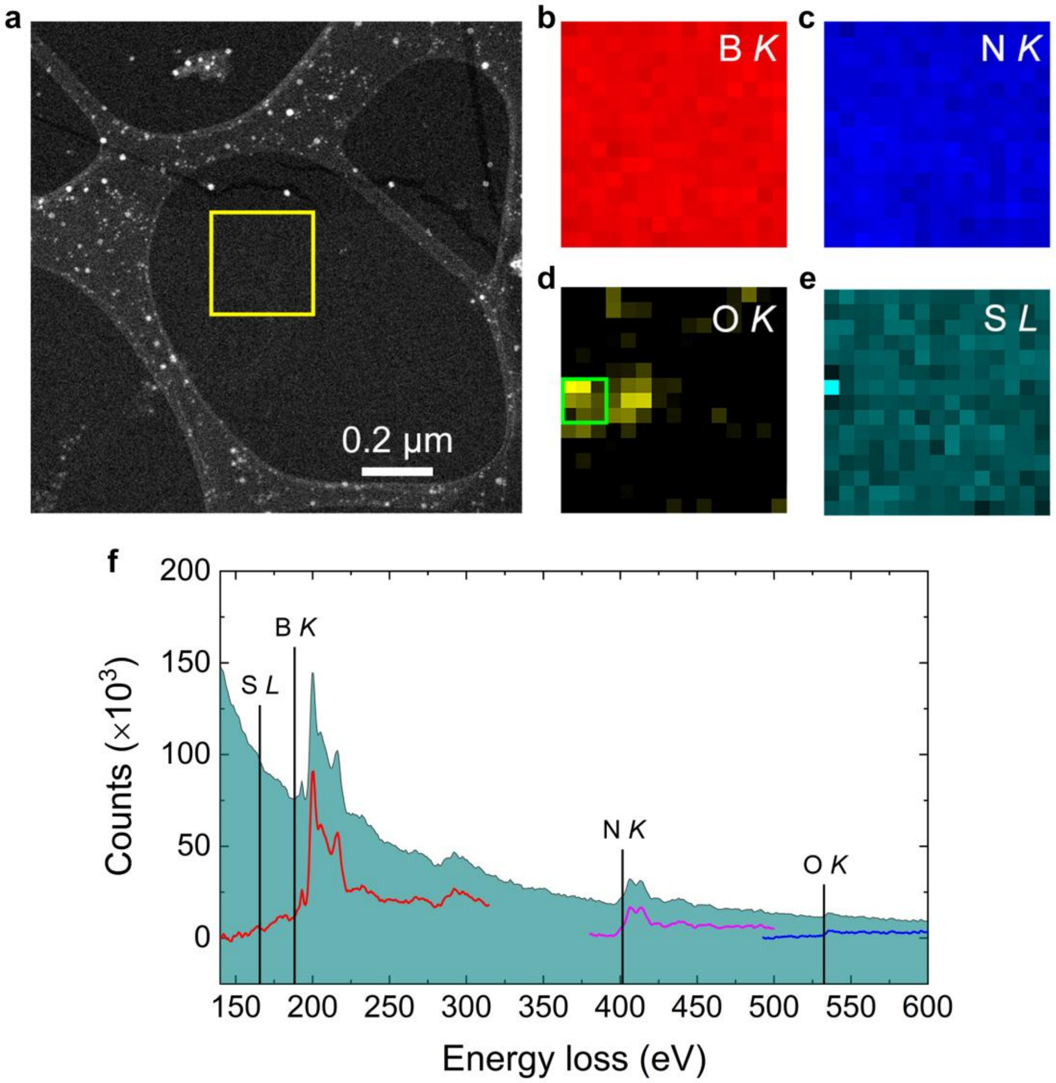

Figure S10. a) ADF-STEM image for the *h*-BN encapsulated WS_2 crystals. b-e) The EELS maps for the boron (b), nitrogen (c), oxygen (d) *K*-edge, and the sulfur (e) *L*-edge measured at the region marked by the yellow square box in (a). f) EELS spectrum measured at the region indicated by the green square box in (e).

S11. Theoretical calculation for EELS analysis

DFT calculation We employee the first-principles density functional theory implemented in the full-potential linearized plane wave (FLAPW) with local orbitals with the ELK code.[9] In the calculation, the local-density approximation exchange correlation functional is adopted and size of basis set, rgkmax, is set to 7.0. $6 \times 6 \times 1$ **k**-point grids are considered for WS_2 (2, 2) supercell with lattice constant of 3.13 Å.[10,11] To consider core-hole excitation state, we perform the pseudo core-hole approach for a local orbital of orbital angular momentum $l = 0$, i.e., for the *K*-shell, in the FPLAPW with deep linearization energy (–25 atomic unit). The pseudo core-hole is made of one of the oxygen nuclei becomes positively charged (+1e) in the all-electron potential together with an additional electron (–1e) simultaneously created at the valence Kohn-Sham orbitals. After the first-principles self-consistent calculation, interactions between all the Kohn-Sham states in the unit-cell and the core-hole become properly demonstrated. The notation of Kohn-Sham Hamiltonian $\widehat{\boldsymbol{H}}_{ks}[\rho]$ and eigenstates $|\boldsymbol{k}, n\rangle$ with core-hole of oxygen *K*-edge can be rewritten as $\widehat{\boldsymbol{H}}_{ks}[\rho; O_i]|\boldsymbol{k}, n; O_i\rangle = E_{n,\boldsymbol{k}}|\boldsymbol{k}, n; O_i\rangle$. The $|\boldsymbol{k}, n; O_i\rangle$ stands for the Kohn-Sham eigenket at a band index n with $\boldsymbol{k}$ momentum. The O_i indicates that the DFT calculation is performed under the Hamiltonian including a core-hole is created at given ith oxygen atom of the oxygen molecule (O_2: $O_{i=1}O_{i=2}$).

EELS calculation and Orbital distribution The EELS can be directly obtained via the following relation,

$$\epsilon_2(\omega; O_i) \sim \sum_{n,m,\boldsymbol{k}} f_{\boldsymbol{k},m}\left(2 - f_{\boldsymbol{k}+\boldsymbol{q},n}\right)\left|\langle \boldsymbol{k} + \boldsymbol{q}, n; O_i|e^{i\boldsymbol{q}\cdot\boldsymbol{r}}|\boldsymbol{k}, m; O_i\rangle\right|^2 \delta\left(E_{n,\boldsymbol{k}+\boldsymbol{q}} - E_{m,\boldsymbol{k}} - \omega\right)$$

The $f_{\boldsymbol{k},n}$ represents occupation number of electrons. In the above relation, the $|\boldsymbol{k}, m; O_i\rangle$ should be an oxygen *K*-shell state of the ith oxygen atom. We define the direction of unit $\boldsymbol{q}$ is approximately aligned along the in-plane cell vectors. The orbital distribution corresponding to each EELS peak can be extracted by following relation, $\rho(\boldsymbol{r}, O_i)_E = \sum_n |\langle \boldsymbol{r}|\boldsymbol{k}, n; O_i\rangle|^2 \delta\left(E - E_{n,\boldsymbol{k}} - E_K\right)$, here E_K is the energy of given core-hole *K*-shell of O_i with constant scissor operator. Note, in the Figure 2(b) of the manuscript, the x-ray absorption for physisorbed oxygen molecules provides identical transition pathways for $\epsilon_2(\omega; O_{i=1})$ and $\epsilon_2(\omega; O_{i=2})$ equivalently so that $\rho(\boldsymbol{r})_E \to \rho(\boldsymbol{r}; O_{i=1})_E$ and $\epsilon_2(\omega) \to \epsilon_2(\omega; O_{i=1})$ are displayed. For the chemisorded oxygen molecules in the Figure 2(c) of the manuscript, however, each oxygen is not equivalent to each other. Thus we show the averaged EELS including two number of cases for core-hole excitations, say, $\epsilon_2(\omega) \to \epsilon_2(\omega; O_{i=1}) + \epsilon_2(\omega; O_{i=2})$ and $\rho(\boldsymbol{r})_E \to \rho(\boldsymbol{r}; O_{i=1})_E$. We will provide the $\rho(\boldsymbol{r}; O_{i=2})_E$ in the Figure S11.

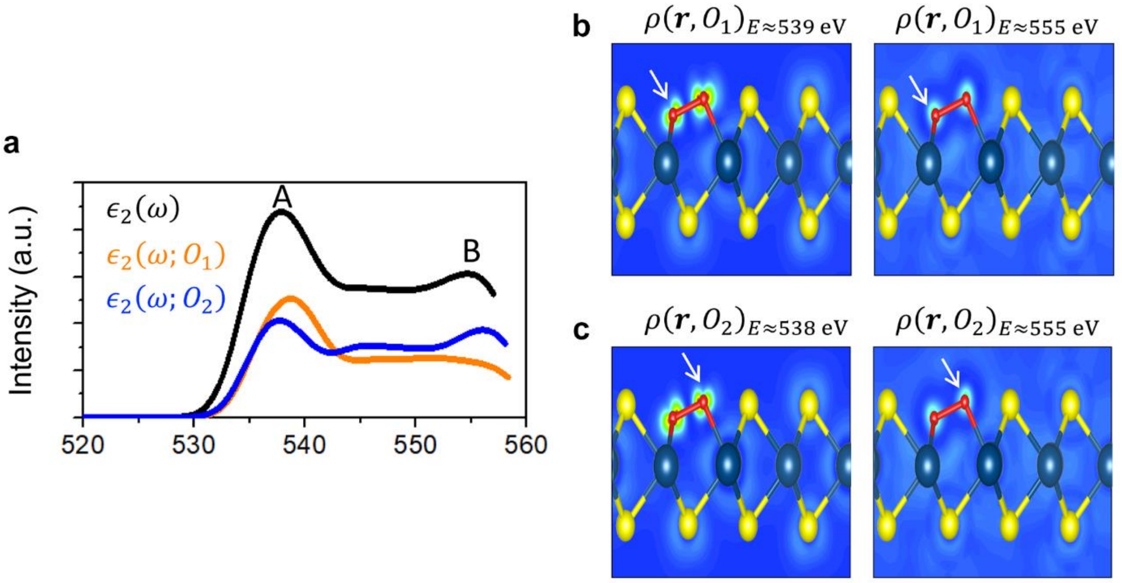

Figure S11. Core-hole dependent EELS spectra and orbital distributions. As long as chemisorbed, the two oxygens are not equivalent to each other and oxygen-dependent core-hole states become distinguishable. Here we assume that the experimental EELS is equally induced from the two kinds of oxygen core-holes. a) core-hole decomposed EELS, $\epsilon_2(\omega) = \epsilon_2(\omega; O_1) + \epsilon_2(\omega; O_2)$, and the $\epsilon_2(\omega)$ is displayed in the Figure 2(c) of the main text. b,c) The $\rho(r, O_i)$ for each peak, A and B, are displayed. White arrows indicate the oxygen atom with core-hole, i.e., $O_{i=1}$ (b) and $O_{i=2}$ (c). The peak A (black) at 538 eV can be decomposed into the two independent peaks at 537.7 eV (blue) and 538.7 eV (yellow). The physical origin of these decomposed peaks is found to be similar as depicted in the (b) and (c). However, the peak B at 555 eV is mostly contributed from that of the $\epsilon_2(\omega; O_2)$.

S12. Power-law for the neutral exciton emission intensity at room temperature

The exciton generation and recombination in the steady-state photoluminescence can be described by the following rate equation:[12]

$$G = \frac{n_X}{\tau_X} + Tn_X n_e + An_X n_e + \gamma n_X^2 \qquad (1)$$

where G is the exciton generation rate, n_X is the neutral exciton density, τ_X is the exciton lifetime, n_e is the electron density, T is the trion formation coefficient, A is the exciton-electron Auger coefficient, and γ is the exciton–exciton annihilation coefficient. Note that the contribution of localized excitons and dark excitons can be neglected by the thermal activation effect at room temperature.[13,14] The exciton-electron Auger ($An_X n_e$) and exciton-exciton annihilation (γn_X^2) processes can be neglected at a low level of excitation, and thus the exciton-to-trion conversion process becomes a dominant nonradiative decay at our experimental conditions. When the neutral exciton and free electron density increase with increasing the excitation power density (for the bare WS_2), the equation (1) can be simplified as the following equation:

$$G \cong Tn_X n_e \qquad (2)$$

When the free electron density increases with the power dependence of P^{μ} ($n_e \sim P^{\mu}$), the neutral exciton density can be written by

$$n_X \cong \frac{G^{1-\mu}}{T} \qquad (3)$$

where the exciton generation rate is proportional to the excitation power ($G \propto P$). The neutral exciton emission intensity for the excitation power can be expressed as the following relation (4), showing that the neutral exciton emission intensity follows the power-law of $P^{1-\mu}$.

$$PL = \frac{n_X}{\tau_X} \cong \frac{G^{1-\mu}}{\tau_X T} \propto P^{1-\mu} \qquad (4)$$

S13. Neutral exciton-to-trion conversion in the bare and *h*-BN encapsulated WS_2

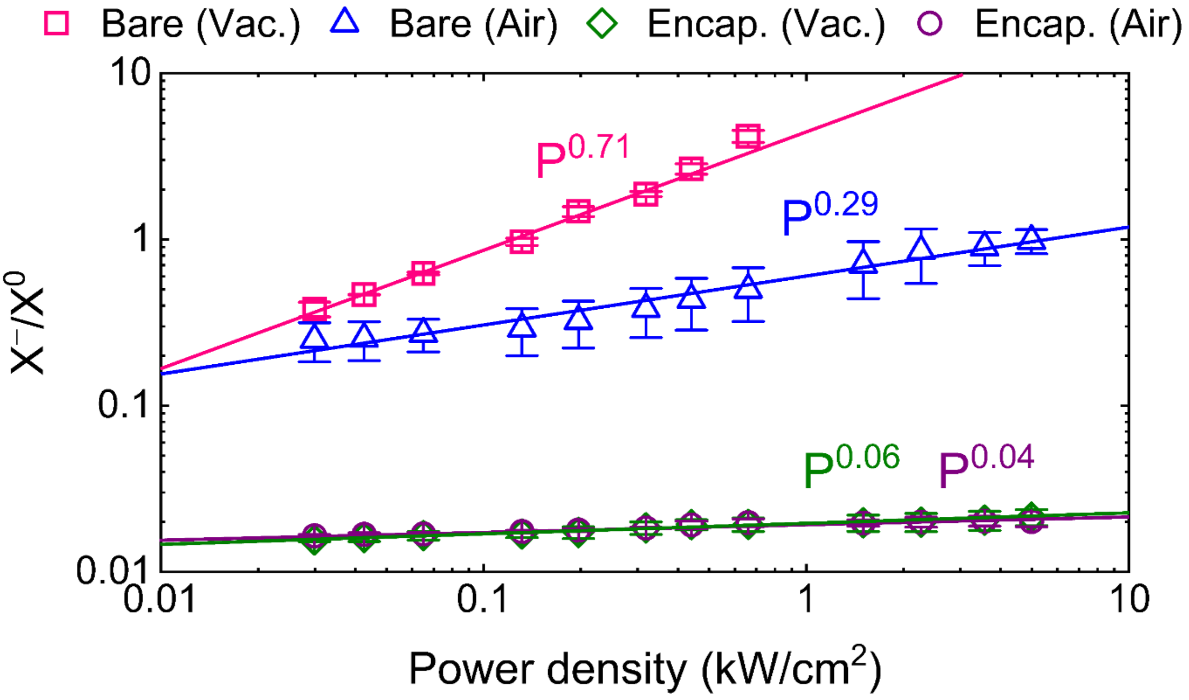

Figure S13. Photoluminescence intensity ratio (X^{-}/X^{0}) of the trion to the neutral exciton with increasing the excitation power density. In a power-law ($X^{-}/X^{0} \propto P^{\mu}$), for the bare WS_2, the neutral excitons are converted to the trions in proportional to P^{μ} with the exponent μ of 0.71 and 0.29 in the ambient vacuum and air conditions with increasing the excitation power, while showing the exponent of 0.06 and 0.04 for the *h*-BN encapsulated WS_2 in the vacuum and air ambient conditions, respectively. As shown in Figures 3(a,b), the exciton emission intensity in the bare and *h*-BN encapsulated WS_2 increases with the exponent $\alpha \cong 1-\mu$ under the vacuum and air conditions, indicating that the neutral exciton-to-trion conversion is the predominant process in the nonradiative decay of the excitons. Thus, Auger recombination process can be neglected under our excitation conditions.

S14. Estimation of the free electron density using mass action law

To determine the free electron density in the bare and h-BN encapsulated WS_2, we used the mass action law describing the formation of the trion (X^-) from the neutral exciton (X^0) and the free electrons (e). The formation of trion can be expressed by the following chemical equation:[15,16]

$$X^0 + e \Leftrightarrow X^- \tag{1}$$

From the chemical equation, the density of each carrier can be related by

$$\frac{n_{X^-}}{n_{X^0}} = \frac{n_e}{K(T)} \tag{2}$$

where n_{X^-}, n_{X^0}, and n_e correspond to the density of trions, neutral excitons, and electrons. $K(T)$ is the temperature-dependent equilibrium constant defined by the following equation:

$$K(T) = \left(\frac{4m_{X^0}m_e}{\pi\hbar^2 m_{X^-}}\right) k_B T exp\left(-\frac{E_b^{X^-}}{k_B T}\right) \tag{3}$$

where m_{X^-}, m_{X^0}, and m_e are the effective mass of the trion, neutral exciton, and electron. The effective mass of the trion and neutral exciton was calculated to be $1.33m_0$ ($m_{X^-} = 2m_e + m_h$) and $0.89m_0$ ($m_{X^0} = m_e + m_h$), where m_0 is the electron mass, m_e ($0.44m_0$) is the effective mass of the electron, m_h ($0.45m_0$) is the effective mass of the hole, k_B is the Boltzmann constant, T is the temperature, and $E_b^{X^-}$ is the trion binding energy (~34 meV).[17,18]

By using the equations (2) and (3), we can obtain the following parameter:

$$\frac{n_{X^0}n_e}{n_{X^-}} = \left(\frac{4m_{X^0}m_e}{\pi\hbar^2 m_{X^-}}\right) k_B T exp\left(-\frac{E_b^{X^-}}{k_B T}\right) = 3.41 \times 10^{12}\ cm^{-2} \tag{4}$$

The intensity weight (I_{X^-}/I_{Total}) of trions in the photoluminescence spectra measured from the bare and h-BN encapsulated WS_2 crystals can be written by the following equation:

$$\frac{I_{X^-}}{I_{total}} = \frac{\gamma_{X^-}n_{X^-}}{\gamma_{X^0}n_{X^0} + \gamma_{X^-}n_{X^-}} = \frac{\frac{\gamma_{X^-}}{\gamma_{X^0}}\frac{n_{X^-}}{n_{X^0}}}{1 + \frac{\gamma_{X^-}}{\gamma_{X^0}}\frac{n_{X^-}}{n_{X^0}}} \tag{5}$$

where γ_{X^0} and γ_{X^-} are the decay rate of neutral excitons and trions, respectively. $\gamma_{X^-}/\gamma_{X^0}$ was estimated by measuring the lifetimes of neutral excitons and trions.

By comparing the parameter (4) and the equation (5), we can obtain the following equation:

$$n_e = 3.41 \times 10^{12} \frac{\left(\frac{\gamma_{X^0}}{\gamma_{X^-}}\right)\left(\frac{I_{X^-}}{I_{total}}\right)}{\left(1 - \frac{I_{X^-}}{I_{total}}\right)}\ [cm^{-2}] \tag{6}$$

From the photoluminescence spectra measured as a function of the excitation power density, we estimated the free electron density in the bare and h-BN encapsulated WS_2 crystals.

S15. Quantitative estimate for the number of desorbed/adsorbed oxygen molecules on sulfur vacancies in-between the vacuum and air envrionments

To investigate a quantitative estimate of the adsorption/desorption of oxygen molecules on WS_2, we fabricated the bare WS_2 capacitor device (Figure S15a), enabling the electrostatic control of electron cencentration as a function of gate voltage (V_g). The gate-voltage-dependent PL measurements were performed at an excitation power density of 0.196 kW/cm^2. As shown in Figures S15c and S15d, the charge neutral poins of the bare WS_2 device were determined at $V_g \cong -14\ V$ and $V_g \cong -9\ V$ under the vacuum (Figure S15c) and air (Figure S15d) ambient conditions, respectively. We calculated the electron densities in the bare WS_2 device at the vacuum and air environments using the equation $\Delta n_{WS_2} = C \times (V_g - V_{neutral})/e$, where Δn_{WS_2} indicates the electron density injected by the gate voltage, C is the capacitance of the used h-BN layer (6.55 × 10^{-4} F·cm^{-2}), V_g is the gate voltage, $V_{neutral}$ is the onset voltage determined by the neutral point, and e is the electronic charge.[19] The electron densities were estimated to be 1.15 × 10^{13} cm^{-2} (vacuum) and 3.69 × 10^{12} cm^{-2} (air) at $V_g = 0\ V$, respectively, while those estimated using mass-action law were 1.11 × 10^{13} cm^{-2} and 2.29 × 10^{12} cm^{-2} at the vacuum and air environments, respectively, showing a good agreement. In-between the vacuum and the air environments, the free electron density induced by the desorption of oxygen molecules is estimated to be 7.77 × 10^{12} cm^{-2} by considering the difference in the free electron densities at the vacuum and air environments. Since an oxygen molecule gains 1.083 electrons from WS_2,[2] the number of desorbed/adsorbed oxygen molecules on the sulfur vacanies were estimated to be 7.17 × 10^{12} cm^{-2}.

On the other hand, we also fabricated the h-BN encapsulated WS_2 capacitor devices (Figure S15b). As shown in Figures S15e and S15f, the charge neutral points of the h-BN encapsulated WS_2 device were determined at $V_g \cong 0.5\ V$ for both the vacuum and air ambient conditions, resulting in the electron densities of 2.05 × 10^{11} cm^{-2}.

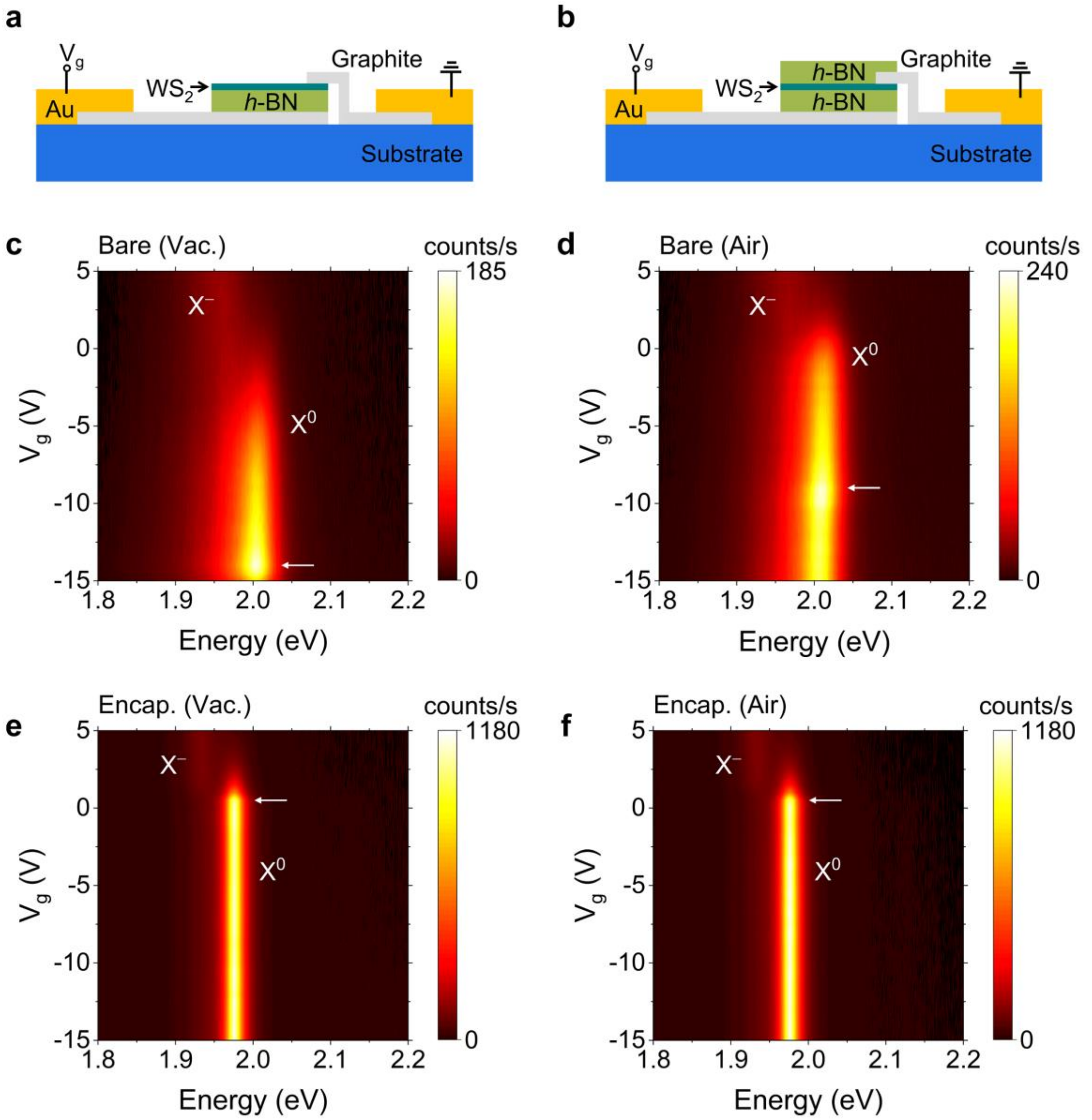

Figure S15. a,b) Schematic illustration for the bare (a) and *h*-BN encapsulated (b) WS_2 capacitor devices. c,d) Gate-voltage-dependent photoluminescence spectral maps for the bare WS_2 device under the vacuum (c) and air (d) ambient conditions. e,f) Gate-voltage-dependent photoluminescence spectral maps for the *h*-BN encapsulated WS_2 device under the vacuum (e) and air (f) ambient conditions.

S16. Photoluminescence decay curves of the neutral excitons under the vacuum ambient condition

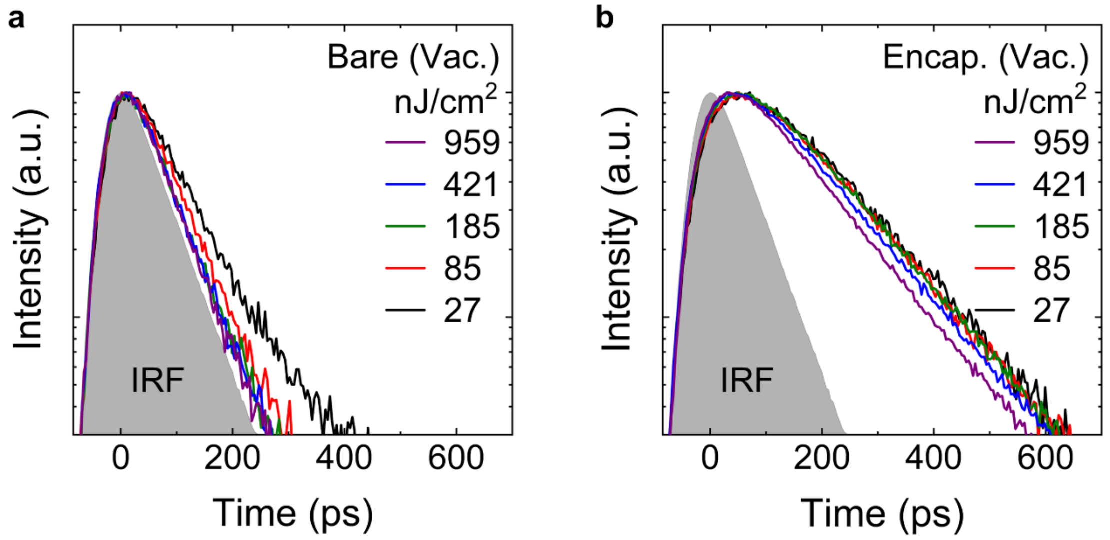

Figure S16. a,b) Photoluminescence decay curves of the neutral excitons measured as a function of the excitation power density for the bare (a) and *h*-BN encapsulated (b) WS_2 crystals under the vacuum ambient condition.

S17. Lifetime of excitons for the bare and *h*-BN encapsulated WS_2 measured as a function of the energy fluence

To extract the lifetime of excitons measured in the vacuum and air ambient conditions for the bare and *h*-BN encapsulated WS_2, the time-resolved photoluminescence decay curves are fitted using an exponential model based on instrument response function (IRF) defined as the following equation:

$$I(t) = \int_{-\infty}^{t} IRF(t')A_1 e^{-\frac{t-t'}{\tau_1}} dt$$

where A_1 and τ_1 are the amplitude and the lifetime for the single exciton component.

Table S1. The lifetime of the neutral excitons measured as a function of the energy fluence for the bare WS_2 under the vacuum and air ambient conditions.

**Energy fluence (nJ/cm^2)**	**Vacuum (bare)**	**Air (bare)**
27	49 ps	74 ps
85	37 ps	63 ps
185	33 ps	57 ps
421	27 ps	47 ps
959	19 ps	43 ps

Table S2. The lifetime of the neutral excitons measured as a function of the energy fluence for the *h*-BN encapsulated WS_2 under the vacuum and air ambient conditions.

**Energy fluence (nJ/cm^2)**	**Vacuum (encap.)**	**Air (encap.)**
27	139 ps	136 ps
85	136 ps	133 ps
185	132 ps	129 ps
421	126 ps	123 ps
959	116 ps	113 ps

S18. Exciton lifetime as a function of free electron density and annihiliation rate constant in the *h*-BN encapsulated WS_2 capacitor devices

Table S3. The lifetime of the neutral excitons in the *h*-BN encapsulated WS_2 capacitor device measured as a function of the gate voltage at a fixed pump fluence of 27 nJ/cm^2.

Gate voltage (V)	Injeced electron density (cm^{-2})	Lifetime (ps)
0.5	2.05×10^{11}	139
0.6	2.46×10^{11}	99
0.7	2.87×10^{11}	62
0.8	3.28×10^{11}	45
0.9	3.69×10^{11}	36

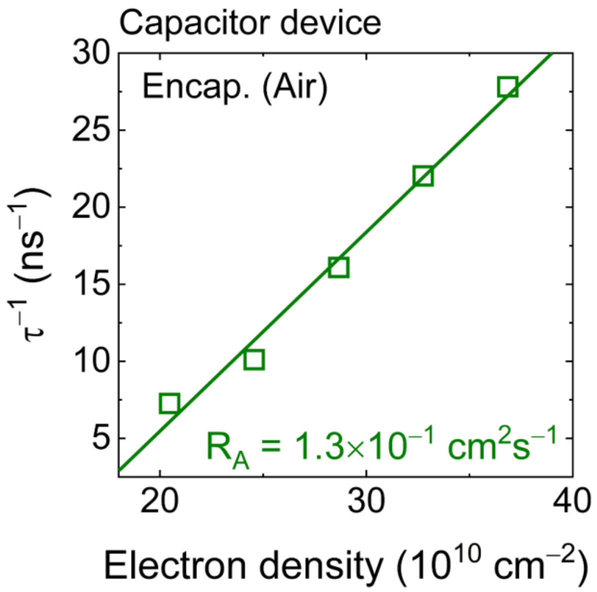

Figure S18. Recombination rate (τ^{-1}) as a function of free electron density for the *h*-BN encapsulated WS_2 capacitor devices. A linear fit for the τ^{-1} results in the exciton annihilation rate constant (R_A) due to the exciton-to-trion conversion process.

S19. Gate-voltage-dependent phtoluminescence decay curves of the neutral excitons in the *h*-BN encapsulated WS_2 capacitor devices

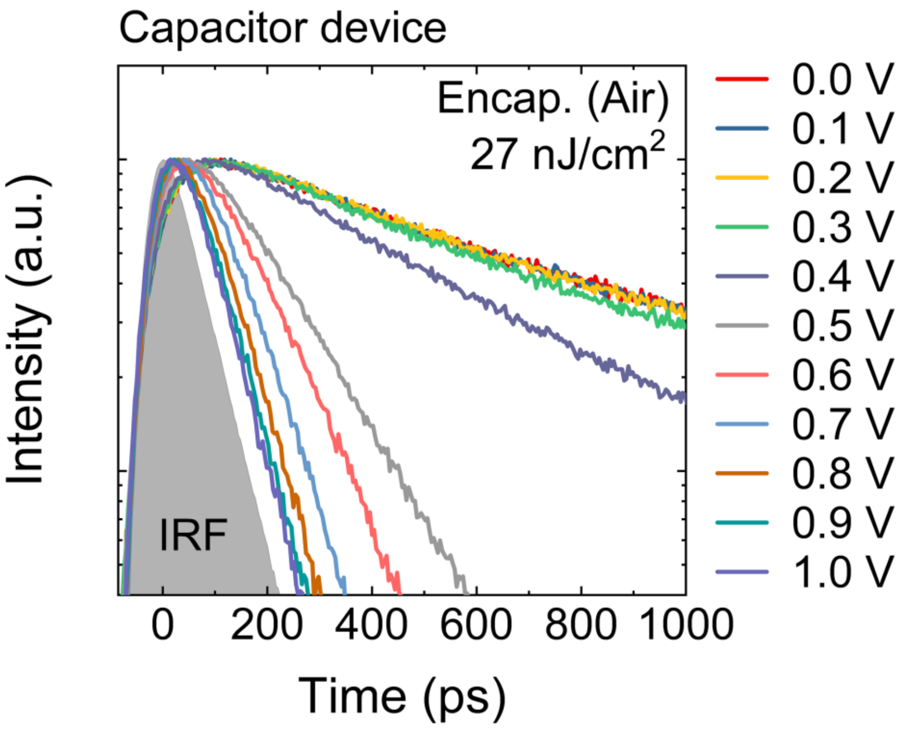

Figure S19. Gate-voltage-dependent photoluminescence decay curves of the neutral excitons in the *h*-BN encapsulated WS_2 capacitor devices under the air ambient condition.

References

[1] H. Nan, Z. Wang, W. Wang, Z. Liang, Y. Lu, Q. Chen, D. He, P. Tan, F. Miao, X. Wang, J. Wang, Z. Ni, *ACS Nano* **2014**, *8*, 5738.

[2] H. Liu, N. Han, J. Zhao, *RSC Adv.* **2015**, *5*, 17572.

[3] D. Ma, B. Ma, Z. Lu, C. He, Y. Tang, Z. Lu, Z. Yang, *Phys. Chem. Chem. Phys.* **2017**, *19* 26022.

[4] T. Kato, T. Kaneko, *ACS Nano* **2016**, *10*, 9687.

[5] G. Plechinger, P. Nagler, J. Kraus, N. Paradiso, C. Strunk, C. Schüller, T. Korn, *Phys. Status Solidi RPL* **2015**, *9*, 457.

[6] A. V. Stier, N. P. Wilson, G. Clark, X. Xu, S. A. Crooker, *Nano Lett.* **2016**, *16*, 7054.

[7] R. Senga, K. Suenaga, *Nat. Commun.* **2015**, *6*, 7943.

[8] J. Pető, T. Ollár, P. Vancsó, Z. I. Popov, G. Z. Magda, G. Dobrik, C. Hwang, P. B. Sorokin, L. Tapasztó, *Nat. Chem.* **2018**, *10*, 1246.

[9] Karl-Franzens-Universität Graz, ELK code, http://elk.sourceforge.net.

[10] J. P. Perdew, W. Y. Wang, *Phys. Rev. B* **1992**, *45*, 13244.

[11] G. B. Liu, W. Y. Shan, Y. Yao, W. Yao, D. Xiao, *Phys. Rev. B* **2013**, *88*, 085433.

[12] D. H. Lien, S. Z. Uddin, M. Yeh, M. Amani, H. Kim, J. W. A. III, E. Yablonovitch, A. Javey, *Science* **2019**, *364*, 468.

[13] T. Godde, D. Schmidt, J. Schmutzler, M. Aßmann, J. Debus, F. Withers, E. M. O. O. Del Pozo-Zamudio, V. Skrypka, K. S. Novoselov, M. Bayer, A. I. Tartakovskii, *Phys. Rev. B* **2016**, *94*, 165301.

[14] X. X. Zhang, Y. You, S. Y. F. Zhao, T. F. Heinz, *Phys. Rev. Lett.* **2015**, *115*, 257403.

[15] J. Siviniant, D. Scalbert, A. V. Kavokin, D. Coquillat, J. P. Lascaray, *Phys. Rev. B* **1999**, *59*, 1602.

[16] S. Mouri, Y. Miyauchi, K. Matsuda, *Nano Lett.* **2013**, *13*, 5944.

[17] A. Ramasubramaniam, *Phys. Rev. B* **2012**, *86*, 115409.

[18] B. Zhu, X. Chen, X. Cui, *Sci. Rep.* **2015**, *5*, 9218.

[19] Y. Shimazaki, I. Schwartz, K. Watanabe, T. Taniguchi, M. Kroner, A. Imamoğlu, *Nature* **2020**, *580*, 472.
